# Emerging Therapies in the Management of Advanced-Stage Gastric Cancer

**DOI:** 10.3389/fphar.2018.00404

**Published:** 2018-09-13

**Authors:** Vivek Kumar, Parita Soni, Mohit Garg, Stephan Kamholz, Abhinav B. Chandra

**Affiliations:** ^1^Brigham and Women's Hospital, Boston, MA, United States; ^2^Maimonides Medical Center, New York, NY, United States; ^3^Oncology, Yuma Regional Medical Center Cancer Center, Yuma, AZ, United States

**Keywords:** andecaliximab, claudiximab, gastric cancer, gastroesophageal cancer, immune checkpoint inhibitor, immunotherapy, napabucasin, The Cancer Genome Atlas

## Abstract

Globally, gastric malignancy contributes to significant cancer-related morbidity and mortality. Despite a recent approval of two targeted agents, trastuzumab and ramucirumab, the treatment options for advanced-stage gastric cancer are limited. Consequently, the overall clinical outcomes for patients with advanced-stage gastric cancer remain poor. Numerous agents that are active against novel targets have been evaluated in the course of randomized trials; however, most have produced disappointing results because of the molecular heterogeneity of gastric cancer. The Cancer Genome Atlas (TCGA) project proposed a new classification system for gastric cancer that includes four different tumor subtypes based on molecular characteristics. This change led to the identification of several distinct and potentially targetable pathways. However, most agents targeting these pathways do not elicit any meaningful clinical benefit when employed for the treatment of advanced-stage gastric cancer. Most advanced-stage gastric cancer trials currently focus on agents that modulate tumor microenvironments and cancer cell stemness. In this review, we summarize data regarding novel compounds that have shown efficacy in early phase studies and show promise as effective therapeutic agents, with special emphasis on those for which phase III trials are either planned or underway.

## Introduction

Gastric/gastroesophageal cancer (GC/GEC) is the fifth most common, and third most deadly, cancer globally. According to the World Health Organization (WHO), 725,000 people worldwide die of stomach cancer, and nearly one million new cases are diagnosed every year^1^. According to the Surveillance, Epidemiology, and End Results (SEER) database, an estimated 97,915 patients with stomach cancer were living in the United States (U.S.) in 2015^2^. Although it is not among the most common malignancies in the U.S. (26,240 new cases estimated in 2018), GC accounts for approximately 11,000 deaths^2^.

In countries where stomach cancer is endemic, community screening has helped increase early diagnosis rates and improve the chances of survival (Rahman et al., 2014). However, in the U.S., approximately 35% of patients have metastatic GC at the time of diagnosis; at this stage, surgery is no longer an option^2^. The patients' 5-year relative survival was 5.2% between 2008 and 2014, compared with 68.1% for patients with localized disease^2^.

Until recently, the treatment options for GC have been limited. However, new molecular targets have been identified based on the molecular classification of GC set forth by The Cancer Genome Atlas (TCGA) network and the Asian Cancer Research Group (ACRG) (Network, 2014; Cristescu et al., 2015). A number of trials were conducted against these novel targets, which include the mechanistic target of rapamycin (mTOR; everolimus), epidermal growth factor receptor (EGFR; panitumumab, cetuximab, and nimotuzumab), the combined target human epidermal growth factor receptor-2 (HER2-neu) and EGFR (lapatinib), mesenchymal-epithelial transition factor (MET; rilotumumab), fibroblast growth factor receptor-2 (FGFR2; AZD4547), and smoothened receptors that are part of the Hedgehog signaling pathway (vismodegib) (Ohtsu et al., 2013; Waddell et al., 2013; Satoh et al., 2014; Cunningham et al., 2015; Jokinen and Koivunen, 2015; Fontana and Smyth, 2016).

**Figure 1:**
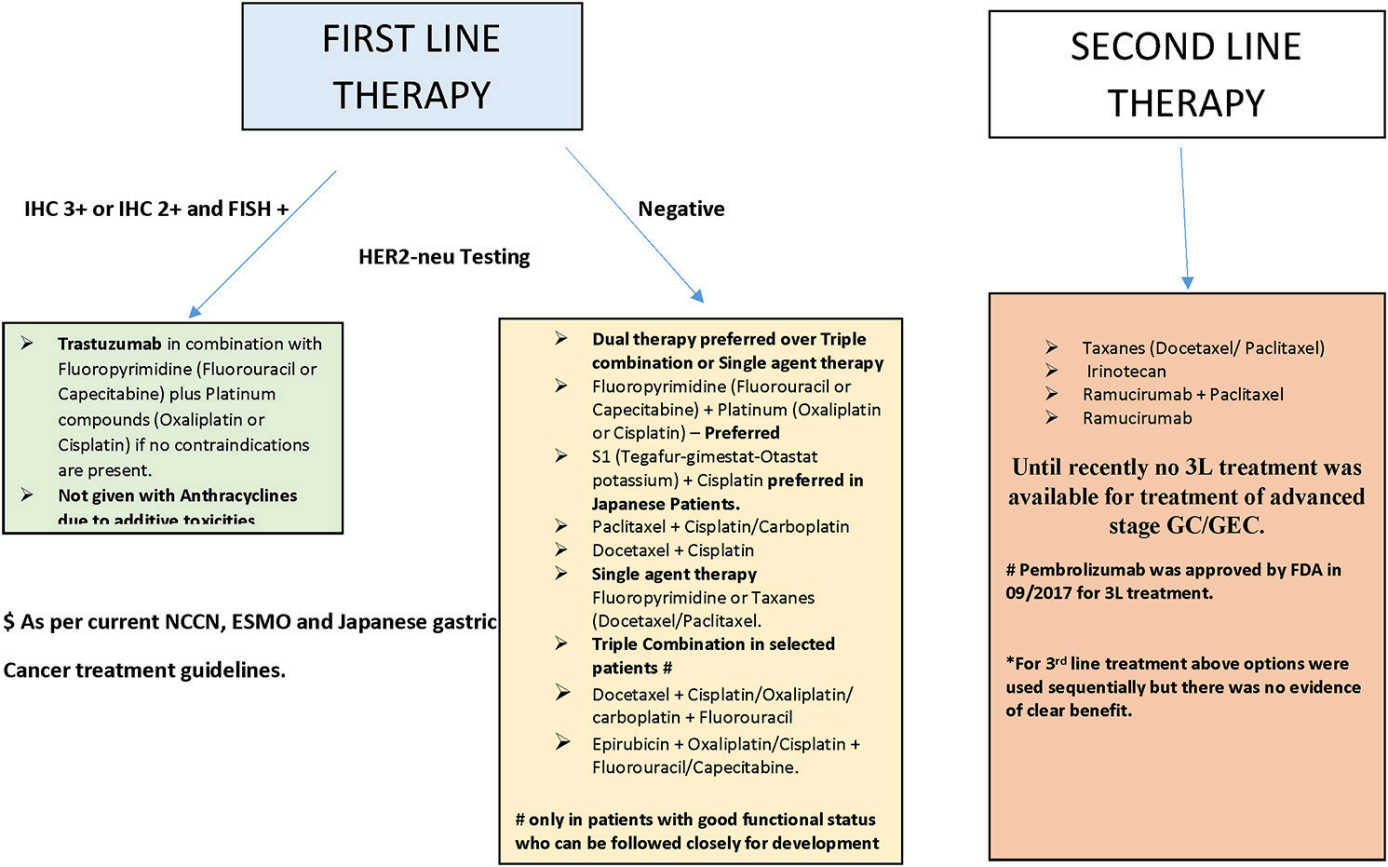
Summary of treatment of advanced stage GC/GEC as recommended by major guidelines (see text).

Food and Drug Administration (FDA) approved two new agents, the HER2-neu antagonist trastuzumab (in 2010) and the vascular endothelial growth factor (VEGF) antagonist ramucirumab (in 2014), for the treatment of advanced-stage GC (Bang et al., 2010; Fuchs et al., 2014). However, the use of these agents is restricted to a specific group of patients with the potential for marginal improvement in their outcome. Most other targeted agents have failed to elicit a survival benefit in patients with advanced-stage GC (Ohtsu et al., 2013; Waddell et al., 2013; Satoh et al., 2014; Cunningham et al., 2015; Jokinen and Koivunen, 2015). The current treatment guidelines for advanced-stage GC issued by major societies, including the National Comprehensive Cancer Network (NCCN), European Society of Medical Oncology (ESMO), and the Japanese Gastric Cancer Association (JGCA) are summarized in Figure 1 (Ajani et al., 2016; Smyth et al., 2016; Association, 2017). Notably, most of these patients suffer cancer progression after several months of treatment with first (1L) and second-line (2L) therapy; however, no established third-line (3L) treatment has been available for these patients until recently (Catalano et al., 2009). Consequently, there is an unmet need for novel targets and targeted agents for the treatment of advanced-stage GC. In the current review, we briefly describe emerging therapies for the management of GC that have shown promise in early-phase studies, with special emphasis on agents for which either phase III trials are planned or have already begun.

## Targeting tumor microenvironments

### Immune checkpoint inhibitors (ICIS)

#### Overview of the immune checkpoints

**Figure 2:**
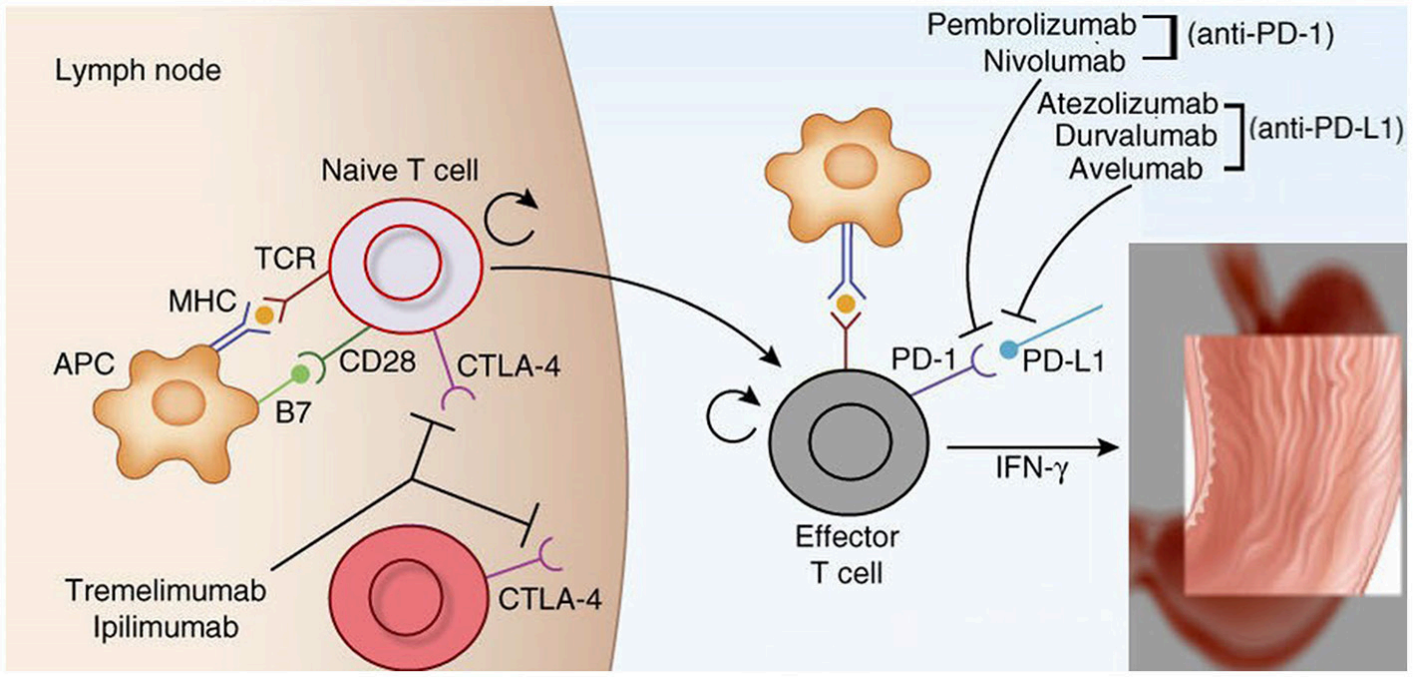
Mechanism of action of cytotoxic lymphocyte associated protein 4 (CTLA-4) and programmed cell death protein 1 (PD-1) and its ligands (PD-L1) (Brahmer et al., 2012; Topalian et al., 2015; Tsai and Hsu, 2017). CTLA-4 plays role in de novo immune stimulation during antigen priming by antigen presenting cells (APCs), macrophages and dendritic cells (DCs). Following antigen exposure, CTLA-4 is expressed on T cells and competes with CD 28 for binding at B7 (B7-1/CD 80 and B7-2/CD 86) on APCs. This generates inhibitory signals for T-cells which shuts off antigen priming by APCs in the tumor draining lymph nodes. CTLA-4 inhibitors namely ipilimumab and tremelimumab restores antigen priming by blocking CTLA-4 on T cells. On the other hand PD-1/PD-L1 axis plays important role in the adaptive resistance by tumors cells against host immune system. PD-1 is expressed on immune cells like T-cells, B-cells and monocytes. The expression of PD-L1 is upregulated on tumor cells and APCs in response to interferon gamma secreted from activated T-cells via activation of JAK2/STAT3 pathway. PDL-2 is also a ligand for PD-1 and is exclusively expressed on DCs. Engagement of PD-L1/L2 by PD-1 inhibits proliferation, migration and effector functions of T-cells. This effect is blunted by PD-1 inhibitors (pembrolizumab and nivolumab) and PD-L1 inhibitors (atezolizumab, durvalumab and avelumab).

Immune checkpoints are molecules that are involved in immune co-inhibitory pathways that shut down the immune system after the clearing of antigens. Important checkpoints include: cytotoxic T-lymphocyte-associated protein-4 (CTLA-4, CD152) and its ligands, CD80 (B7-1) and CD86 (B7-2); indoleamine-pyrrole 2,3-dioxygenase (IDO); lymphocyte-activation gene 3 (LAG-3, also known as CD223); programmed cell death protein-1 (PD-1 or CD278) and its ligands, PD-L1 (B7-H1 or CD274) and PD-L2 (B7-DC or CD273); T-cell immunoglobulin and mucin-domain-containing-3 (TIM-3), and its ligand galectin-9 (Soliman et al., 2010; Tsai and Hsu, 2017). Among these checkpoints, the two most important targets of immunotherapy are CTLA-4 and the PD-1/PD-L1 axis. Figure 2 describes their mechanisms of action in detail.

#### Rationale for the use of ICIS in the treatment of GC/GEC

Immune checkpoints maintain self-tolerance and prevent collateral damage when the immune system attacks foreign cells, and are essential for checking excessive stimulation of the immune system and preventing autoimmunity (Topalian et al., 2015). However, these checkpoints may be counteractively used by tumor cells to evade host immunosurveillance (Brahmer et al., 2012) and escape immune destruction. Because of that, inhibition of checkpoints by ICIs helps restore host immunity against tumor cells (Brahmer et al., 2012). Indeed, treatment with ICIs has elicited sustained responses in individual patients with advanced-stage cancer. Thus, these agents constitute the most promising new therapeutic options for patients with GC (Kelly, 2017).

GC was reclassified into four subtypes based on molecular phenotypes identified by utilizing integrative genomics (Network, 2014). Among these, the Epstein-Barr virus (EBV)-related and microsatellite instability (MSI) subtypes exhibit immune signatures and tumor microenvironments amenable to treatment with ICIs (Kelly, 2017). The EBV subtype is characterized by a high prevalence of DNA hypermethylation and amplification of *CD274* and *PGD1LG2* (which encode PD-L1 and PD-L2, respectively) (Network, 2014). This subtype has been observed by TCGA network analysis in up to 9% of patients. MSI subtypes, which constitute 22% of GCs, have a high mutational burden and neoantigen presentation with tumor infiltrating lymphocytes (TILs), dendritic cells (DCs), and macrophages.

The melanoma microenvironment has been classified into four subtypes based on the mechanisms by which tumor cells evade host immunosurveillance (Teng et al., 2015). Type 1 is most responsive to single-agent ICI therapy, and is characterized by the presence of both PD-L1 and TILs in the tumor microenvironment. This suggests that adaptive immune resistance by tumor cells is associated with the up-regulation of PD-L1 in immune cells, leading to T-cell anergy after binding PD1. This type of microenvironment has not yet been described in GC. However, enhanced PD-L1 expression has been demonstrated in EBV-positive and MIS subtypes of GC.

PD-L1 is overexpressed in up to 42% of GCs (Wu et al., 2006). However, there is a great variation in the PD-L1 positivity rate, between 12.3 and 64%, as reported in various studies (Table 1). PD-L1 expression is particularly enriched in the EBV and MSI subtypes. In the EBV subtype, 50% of tumors and 94% of immune cells stain positive for PD-L1, while in the MSI subtype, PD-L1 expression is found in 33% of tumors and 45% of immune cells (Derks et al., 2016). With the exception of EBV and MSI subtypes, PD-L1 expression is uncommon in GC cells. In contrast with other cancers and EBV-positive GC, PD-L1 expression occurs in immune cells at the tumor margins in EBV-negative GC, while more diffuse infiltration has been observed in the former (Derks et al., 2016). Moreover, in GC, elevated levels of PD-L1 are noted in the stroma, whereas, in other cancers, such elevated levels of PD-L1 are noted in the membranes (Derks et al., 2015; Muro et al., 2016; Thompson et al., 2016). The implications of this differential expression are not yet clear; however, this expression pattern may be relevant to the development of biomarkers or to the relatively lower efficacy of ICIs in GCs compared with that in melanoma or lung cancer (Kelly, 2017).

**Table 1:** Summary of selected clinical trials on emerging therapies for the treatment of gastric cancer.

**Study name (status)**	**Setting and design**	**Results and comments**
**KEYNOTE-012** (Muro et al., 2016) 2016* NCT 01848834 (completed) Pembrolizumab @10 mg/kg once Q2W for 24 months or until criteria to stop therapy were met. **Primary end points**: Safety and tolerability Overall response	PD-L1 + patients with chemorefractory/chemo intolerant GC/GEC Patients with origin in East Asia (19) and rest of the world (20) Phase Ib	PD-L1+rate = 65/162 (40%), 39 enrolled in the trial. 67% heavily pretreated with ≥2 lines of chemotherapy. 26 (67%) AEs, grade 3 and 4 AEs in 5 (13%) patients. Fatigue 7 (18%), loss of appetite 5 (13%), hypothyroidism 5 (13%), pruritus 5 (13%), and arthralgia 4 (10%). IRAEs in 3 (8%) Asian patients and 6 (15%) in the rest of the world arm. Overall ORR: Central review 8 (22%; 95% CI: 10-39); investigator review 13 (33%, 19–50). Median DOR: 40 weeks. CR None. 33 (85%) patients discontinued treatment after 10.8 months. (mostly due to disease progression) PFS= 1.9 months. OS= 11.4 months. 4/24 (17%) MSI positive. Of 4, 2(50%) PR.
**KEYNOTE-059 cohor t(Fuchs et al.**, **2017****)** 2017 NCT 02335411 (completed) Pembrolizumab Pembrolizumab 200 mg Q3W up to 2 y or up to disease progression or until criteria to discontinue were met. **Primary end points**: ORR, safety, and tolerability.	3rd and 4th line therapy in PD-L1 unselected patients with advanced GC/GEC Phase II	259 patients. PD-L1 positivity rate of 57.1%. Overall ORR: 11.2% (95% CI: 7.6–15.7); CR = 1.9%. PR = 9.3%. ORR (%) was 14.9 (9.4–22.1) in 3L pts and 7.2 (3.3-13.2) in 4L+ Median DOR: 8.1 months. ORR: 15.5 (10.1–22.4) in PD-L1 + and 5.5 (2–11.6) in PD-L1 negative. ORR in the 3L+, PD-L1 +: 21.3% (95% CI: 12.7–32.3) and CR 4.0% (95% CI: 0.8–11.2). Grade 3-5 AEs = 43 (16.6%), deaths = 2.
**KEYNOTE-059 cohort 2** (Bang et al., 2017a) NCT 02335411 (ongoing) Pembrolizumab Pembrolizumab 200 mg Q3W plus cisplatin 80 mg/m^2^ for 6 cycles and 5-FU 800 mg/m^2^ (or capecitabine 1,000 mg/m^2^ in Japan) Q3W for up to 2 years or until the termination criteria were met. **Primary end points:** Safety and tolerability	1st line treatment in combination with standard of care therapy in PD-L1 unselected treatment naïve metastatic/recurrent GC/GEC Phase II	25 patients; PD-L1 positivity rate of 64% (16/25). At 12.2 months; 84% discontinued treatment. Grade 3-4 AEs = 76%. ORR: 60% (95% CI, 38.7-78.9). SD = 32%. PD = 4%. ORR: 68.8% (95% CI, 41.3-89.0) in PDL-1+ and 37.5% (8.5-75.5) in PD-L1– patients. Overall median DOR = 4.6, 4.6 months in PD-L1+ patients: 4.6 months and PD-L1–: 5.4 months. PFS = 6.6 months; OS = was 13.8 months.
**KEYNOTE-059 cohort 3** (Catenacci Daniel et al., 2017) NCT 02335411 (ongoing) Pembrolizumab Pembrolizumab 200 mg every 3 weeks for up to 2 years or until the termination criteria were met. **Primary end points:** ORR, Safety and tolerability	1st line treatment as single agent in PD-L1 +, treatment naïve metastatic/recurrent GC/GEC Phase II	31 patients. Majority M1 stage (83.9%). ORR: 25.8% (95% CI, 11.9–44.6), CR: 3.2% of patients. Median DOR not reached (range, 2.1–13.7+). Median PFS: 3.3 months. Median OS not reached after 14.5 months (9.2-ne) OS of 61.7% at 1 year and 73% at 6 months. Any grade AEs: 24 (77.4%), and 7 (22.6%) grade 3–5 events. 1 deaths
**NCT 02689284 (Catenacci et al.**, **2018****)** In phase 1b Margetuximab @ 10 and 15 mg/kg with In phase 2 patients received Margetuximab @ 15 mg/kg. Pembrolizumab 200 mg of pembrolizumab Q3W in both the phases. **(Ongoing)**	2nd line treatment in relapsed or advanced HER2+ GC/GEC with failed treatment with trastuzumab plus chemotherapy Phase Ib/II	51 patients, (29 North America/22 Asia) PD-L1 unselected 53% (27/51) response Overall ORR: 18% (6 confirmed and 3 unconfirmed PR), SD in 19 (37%), DCR 55% ORR (GC vs. GEC): (32% vs. 4%), DCR (72% vs. 38%) and median PFS (5.5 vs. 1.4 months) higher in GC vs. GEC. Grade ≥3: 11.9%. Fatigue most common AE (of any grade in 14.9%).
**ONO-4538-12** (Kang et al., 2017a) 2017 (ATTRACTION-2) (ongoing) Nivolumab Nivolumab @ 3 mg/kg or placebo intravenously every 2 weeks **Primary endpoint:** OS	3rd line treatment in PD-L1 unselected Unresectable/recurrent GC/GEC in patients who received ≥2 lines of chemotherapy Asian patients Phase III	493 patients. 2:1 ratio to nivolumab (*n* = 330) or placebo (*n* = 163) arms. PD-L1 + rate 13.5% (26/192); 12.3% (16/130) in the nivolumab and 16·1% (10/62) in the placebo. Follow-up time 8·87 months in the nivolumab group and 8·59 months in the placebo group; 290 (87·9%) patients in the nivolumab arm and 158 (98·1%) patients in the placebo group discontinued treatment. Median OS: nivolumab 5.32 months vs. 4.14 in the placebo arm *p* < 0.0001. OS at 12 months was 26.6% (95% CI: 21.1–32.4) in nivolumab arm vs. 10.9% (6.2–17) placebo group. ORR in the nivolumab arm 11.2 (95% CI: 7.7–15.6) vs. 0% (0.0–2.8) in placebo arm. Median PFS with nivolumab was 1.61 and 1·45 months in the placebo arm; HR 0·60 (95% CI: 0.49–0.75; p < 0.0001). Nivolumab reduced mortality by 37% (HR 0.63 *p* < 0.0001). DOR was 9.53 months. Any grade AEs (42.7% vs. 26.7%) and grade 3/4 AEs (10.3% vs. 4.3%) higher in nivolumab arm but the difference not statistically significant. AEs related deaths: 5 (2%) in nivolumab arm and 2 (1%) in the placebo arm.
**CheckMate-032** (Janjigian et al., 2017) 2017 NCT 1928394 (Completed) Nivolumab + Ipilimumab Nivolumab 3 mg/kg every 2 week (N3) Nivolumab 1 mg/kg plus Ipilimumab 3 mg/kg Q3 weeks (N1+I3) Nivolumab 3 mg/kg with Ipilimumab 1 mg/kg Q3 weeks (N3+I1). **Primary end point:** ORR	Heavily pretreated PD-L1 unselected chemorefractory advanced stage GC/GEC Western patients Phase I/II	166 patients. (79% were treated with ≥ 2 lines of chemotherapy) into 3 groups: (N3, *n* = 59), (N1+I3, *n* = 49), (N3+I1, *n* = 52). PD-L1 positivity rate of 24%. ORR: N3– 12%, N1+I3 −24%, and N3 +I1 −8%. In PD-L1 +, ORR: N3-19%, N1+I3 −40% and N3+I1 −23%. In PD-L1 –, ORR: N3-12%, N1 +I3-22%, and N3+I1 −0%. Overall, 1-year OS: 9% in N3, 35% in N1+I3, and 24% in N3+I1. PD-L1 +, 1-year OS: 34% in N3, 50% in N1+I3, and 23% in N3+I1. Grade 3/4 AEs in > 10%: diarrhea (2% in N3, 14% in N1+I3, and 2% in N3+I1), elevation of ALT (3% in N3, 14% in N1+I3, and 4% in N3+I1) and AST (5% in N3, 10% in N1+I3, and 2% in N3+I1).
**JAVELIN solid tumor (Chung et al.**, **2016****)** NCT01772004 2016 Avelumab @10 mg/kg IV Q2W **Primary end points:** AEs, ORR and PFS	First-line Mn or 2L therapy in PD-L1 unselected patients advanced GC/GEC Phase Ib	151 (62 patients as 2L; 89 patients as Mn) and follow up time 49 weeks. PD-L1+: 49% 74 (22/62 2L, 52/89 Mn) Any grade AEs: 89 (58.9%); infusion reaction 19 (12.6%) and fatigue in 16 (10.6%). Grade ≥3 AEs 15 (9.9%); fatigue, asthenia, increased GGT, thrombocytopenia, and anemia occurred in 2 patients each (1.3%). 1 Fatal AE (hepatic failure/autoimmune hepatitis). Overall ORR as 2L: 6/62 (9.7%), all PRs; Mn: 8/89 (9.0%), 2 CRs: 6 PRs. Overall DCR: 29% in 2L and 57.3% in Mn. PFS: 6 wks in 2L and 12 wks in Mn In 2L, ORR (%): 18.2 in PD-L1 + vs. 9.1 in PD-L1 –.PFS (weeks) 6.3 PD-L1 + and 10.4 in PD-L1 –. In Mn, ORR (%): 10 in PD-L1 + vs. 3.1 in PD-L1 –. PFS (weeks) 17.6 PD-L1 + and 11.6 in PD-L1 –.
**JAVELIN dose expansion study 2016 (Nishina et al.**, **2016****)** (NCT01943461) Avelumab 10 mg/kg Q2W IV until study criteria to stop therapy were met. **Primary end points:** AEs, ORR and PFS	Monotherapy in Japanese patients with advanced stage PD-L1 unselected GC/GEC Phase Ib	20 patients. Follow-up time: 6 months PD-L1 + rate 5/19 (26.3%). Any grade AEs: 18/20 pts (90%); 1 patient (5%) grade 3 AE. ORR: 15.0% (all PRs) and DCR: 65.0%. ORR (%) was 40 in PD-L1 + compared to 7.1 in PD-L1 –. Median PFS (weeks): 12.3 for PD-L1 + and 11.1 for PD-L1 –. PFS (%) 60.0 and 32.1 at 12 weeks for PD-L1 + and PD-L1 –, respectively.
NCT 00909025 (completed) Claudiximab (formerly IMAB 362) Escalating doses were administered to 3-6 patients to determine the maximum tolerated dose (MTD).	Phase I Metastatic GC/GEC	15 patients. Dose escalation study. Concluded that dose up to 1 gm/m^2^ was safe in GC.
**PILOT (Sahin et al.**, **2015****)** NCT 01771774 (completed) Arm 1: Claudiximab (800 mg/m^2^ in cycle 1, followed by 600 mg/m^2^ Q3W) plus ZA (4 mg, IV, day 1 of cycle 1 and 3) Arm 2: Claudiximab, ZA plus IL-2 (1 × 106 IU, s.c., days 1–3 of cycle 1 and 3) Arm 3: Claudiximab, ZA plus IL-2 (3 × 106 IU s.c., days 1–3 of cycle 1 and 3) Arm 4: Only Claudiximab **Primary end point:** immunomodulatory Biomarkers	Monotherapy and in combination with ZA and interleukin-2 for immunomodulation I chemo-refractory disease Phase I	28 patients Primary endpoints have not been reported 26 patients ≥1 AEs, 13 patients had ≥1 special AEs. Majority AEs grade 1–3; nausea and vomiting in 16 (2 patients grade 3) and 15 (2 patients grade 3) patients, respectively. DCR: 55% (10 stable and 1 PR). PFS: 12.7 weeks. OS: 40 weeks.
**MONO (Trarbach et al.**, **2014****) NCT 01197885 (completed)** IMAB362 at 300 or 600 mg/m^2^ Q2W PFS and AEs	Monotherapy in Metastatic, refractory or recurrent CLDN18.2 + GEC/GC Phase IIa	54 patients. Claudiximab at doses of 300 mg/m^2^ (4 patients) and 600 mg/m^2^ (50 patients). PFS: 102 days. RR: 10%, DCR: 30% (PR: 4 and SD: 8). Nausea (31/54) and vomiting (27/54) most common AEs. 8 and 13 patients experienced grade 3 nausea and vomiting respectively. Drug pharmacokinetics supported 3-weekly IV dosing.
**FAST** (Schuler et al., 2016) NCT 01630083 (completed) Claudiximab + standard chemotherapy epirubicin/oxaliplatin/capecitabine (EOX) Arm 1 EOX Arm 2 EOX plus claudiximab @ 800 mg/m^2^ loading dose, followed by 600 mg/m^2^ on day 1 Q3W. Arm 3: higher-dose claudiximab 1,000 mg/m^2^ with EOX. **Primary end point:** PFS	As 1st line treatment in combination with standard chemotherapy (EOX) for metastatic/recurrent CLDN18.2 + (defined as expression of ≥2+ in ≥40% tumor cells) GC/GEC Phase IIb	686 patients, 334 (48%) met study criteria of CLDN18.2 expression on initial tumor screening, and final data were presented for 246 patients (only for Arms 1 and 2 below). 161 patients (80% gastric; 44% diffuse, 33% intestinal): 84 in arm 1, 77 in arm 2. 85 in the ^+^. Claudiximab plus EOX vs. EOX; PFS: 7.9 vs. 4.8 months; HR 0.47. OS: 13.2 vs. 8.4 months; HR 0.51 and ORR: 43% vs. 28%. The response was better in patients with 70% or more of the tumor cells expressing CLDN18.2, claudiximab improved the median PFS from 5.6 months for EOX alone to 7.2 months (HR = 0.36, *P* < 0.0005), and OS improved from 9 to 16.7 months (HR = 0.45, *P* < 0 .0005). Vomiting (34.5% grade 1/2 and 3.6% with grade 3/4 in the EOX arm vs. 55.8% grade 1/2 and 10.4% with grade 3/4 in claudiximab plus EOX arm) Neutropenia (21.4% grade 1/2 and 21.4% grade 3/4 in EOX arm vs. 23.4 % grade 1/2 and 32.5% grade 3/4 in claudiximab arm). Other AEs: anemia and diarrhea of grade 1/2 severity in both the arms.
**NCT01803282 (Shah et al.**, **2017b****)** mFOLFOX with Andecaliximab (GS-5745) 800 mg iv Q2W AEs, OS, PFS and MMP-9 inhibition reported.	Andecaliximab In combination with mFOLFOX in metastatic GC Phase Ib	40 patients. 29 chemotherapy naïve (First-line) Most common any grade AEs, nausea (62.5%), fatigue (60%), diarrhea (45%), and peripheral neuropathy (45%). Grade ≥3 AEs: neutropenia (20 %). Overall, PFS: 7.8 months, DOR: 10.1 months and ORR: 50%. As 1L, PFS: 12 months, DOR: 10.6 months and ORR: 55.2%. Collagen neoepitope levels (MMP 9) trended down with continuous therapy suggesting a therapy related effect.
**JapicCTI-14242072 (Shitara et al.**, **2015****)** BBI608; 480 mg bid. in combination with PTX; 80 mg/m^2^ Q1W, on day 1, 8, and 15 of Q4W cycle Safety, tolerability, PK, and efficacy in combination with weekly PTX	Pretreated unresectable or recurrent disease Phase I	6 patients. No dose limiting toxicities were observed. Grade 1 diarrhea 100% and grade 1 anorexia in 2 (33.3). PR: 2 patients (33.3%). In one patient PR lasted for > 7.5 months. SD: 2 patients. (2.8 months) or non CR/PD (7.5 months). Similar PK profiles among patients with (*n* = 3 [50%]) or without (*n* = 3 [50%]) gastrectomy.
**NCT 01325441 (Hitron et al.**, **2014****)** BBI608 in 3 doses: 200 mg BID, 400 mg BID, 500 mg BID with paclitaxel (80 mg/m^2^ weekly; 3 of every 4 weeks) Dose escalation study	Refractory disease (in combination with paclitaxel) Phase Ib/II	In phase Ib, of 24 patients, 5 with refractory GEC/GC PR: 2 (with 48% and 45% regressions), 1 SD with 25% regression, and 2 prolonged SD ≥ 24 wks. Dose of 500 mg BID was determined and tested in phase II (see below).
**NCT 01325441 (Becerra et al.**, **2015****)** BBI608 orally at 480 mg or 500 mg BID with paclitaxel 80 mg/m^2^ weekly 3 of every 4 weeks ORR, DCR and PFS	Phase II of the above study.	46 (87% Caucasian) 10 (22%) received 1, 16 (35%) 2, and 20 (43%) ≥3 or more lines of chemotherapy. AEs: Grade 1-2 GI symptoms. Grade 3 AEs: vomiting (10%), diarrhea ≥5 days (7%), fatigue (7%), and abdominal cramps, nausea, dehydration (2% each). In Taxane naïve 20 patients, ORR: 31% (5/16), DCR: 75% (12/16); PFS: 20.6 weeks and OS: 39.3 weeks In taxane resistant 26 patients, ORR: 11% (2/19), and DCR: 68% (13/19); PFS: 12.6 weeks and OS: 33.1 weeks ORR: 50% (3/6) and DCR: 83% (4/6) among taxane naïve patients who were treated with only 1 prior line of chemotherapy.
**JACOB (Tabernero et al.**, **2017****) (NCT01774786)** Placebo + Trastuzumab (T) + CT (cisplatin/fluoropyrimidine) or Pertuzumab (P) + T + CT. P and T Q3W. P @ 840 mg, T @ 8 mg/kg loading and 6 mg/kg maintenance doses). OS. Secondary endpoints PFS and AEs	Phase III study in HER2 + metastatic GC/GEC.	388 patients in Pertuzumab arm and 390 placebo arm. OS = 17.5 months in pertuzumab arm vs. 14.2 months in the placebo arm; HR 0.84. PFS 8.5 months in pertuzumab vs. 7 months in placebo arm. More Diarrhea in (61.6%) in pertuzumab arm vs. placebo (35.1%). Addition of pertuzumab to trastuzumab + chemotherapy failed to demonstrate statistically significant improvement in OS. Net gain of 3 months in OS was reported.

AE Adverse event; CR complete remission; DOR Duration of response (time from documentation of tumor response to disease progression); Disease control rate (DCR) = (CRR+ PR + SD) GC Gastric cancer; GEC gastroesophageal Cancer; PFS progression free survival; Overall response rate (ORR) = (CR+PR); OS overall survival; PR partial remission; SD stable disease;

Drug developers use different immunohistochemistry (IHC) methods for PD-L1 staining (Tran et al., 2017). While concordance between antibody assays for the detection of PD-L1 is high for nivolumab [Dako, 28-8 antibody (31)(31)(31)(31)], pembrolizumab (Dako, 22c3 Ab), and durvalumab (Ventana, SP263 Ab), intratumor and intertumor variability, as well as interobserver variations, remain limiting factors (Gaule et al., 2017; Tran et al., 2017). In addition, there is a lack of harmonization for the cut-off thresholds of staining assays. The cut-off thresholds for PD-L1 positivity in most nivolumab trials are as follows: 0–1% (negative), 1–5% (weak), 5–10% (medium), and >10% (strong). By contrast, the cut-off thresholds for pembrolizumab are 0–1% (negative), 1–50% (medium), and >50% (strong) (Tran et al., 2017). To date, there is no consensus regarding PD-L1 expression and prognosis of patients with GC, as some studies have suggested improved outcomes, while others have not (Böger et al., 2016; Eto et al., 2016; Muro et al., 2016; Schlößer et al., 2016).

Other useful indicators suggestive of a role for ICIs in GC/GEC include the presence of chronic inflammation associated with the Barrett's esophagus and defective mismatch repair genes (*MMR*), which are noted in up to 21% of patients with GC (Moons et al., 2005; Network, 2014; Giampieri et al., 2017). Thus, infection, chronic inflammation, and involvement of the immune system make GC tumor microenvironments responsive to ICI treatment (Kelly, 2017).

#### Clinical evidence for ICIs in GC

##### PD-1 inhibitors (pembrolizumab and nivolumab)

The KEYNOTE 012 trial tested the tolerability and safety of single-agent pembrolizumab as 2L treatment in patients with GC (Muro et al., 2016; Table 1). This investigation was a phase Ib trial that included only PD-L1–positive advanced-stage/recurrent GC/GEC, with equal representation of Asian and non-Asian patients. That is important because of the differences in the incidence and age at initial diagnosis of GC in these two populations. The response to therapy is also different in these two populations, as noticed in an earlier trial (Van Cutsem et al., 2012). Further, tumors from non-Asian individuals exhibit stronger immune signatures, including higher TILs and CTLA-4 signaling, whereas the immunosuppressive regulatory T-cell (Treg) marker FOXP3 is more highly expressed in Asians (Lin et al., 2015). These data suggest enhanced response to ICIs in non-Asians; however, in the cited study, there was no difference in the outcome in these two groups. This unexpected finding may be attributed to the small sample size in each arm because of which the study lacked sufficient power to detect a small difference in survival. Further research involving larger sample sizes is warranted. Although the cited study failed to meet the primary endpoint of the overall response rate (ORR) of 31%, the ORR was 22%, and all patients showed partial remission (PR). A direct comparison with earlier chemotherapy trials is not feasible because of the differences in the characteristics of the study population. Nevertheless, the median overall survival (OS) of 11.4 months with pembrolizumab in this study is comparable with the 9–10 months reported in trials on combination chemotherapy, and a definite improvement over the OS of 4–5 months achieved by single-agent chemotherapy in a 2L setting (Catalano et al., 2009; Wilke et al., 2014). The adverse events were similar to those for pembrolizumab trials in other cancers. The results from this study indicate the feasibility and safety of pembrolizumab in heavily pretreated advanced-stage GC/GEC **[NCT 01848834]**.

The role of pembrolizumab in advanced-stage GC/GEC was further explored in a large multicohort phase II trial, KEYNOTE 059. Cohort 1 was designed to assess the safety and efficacy of single agent pembrolizumab as 3L therapy (Fuchs et al., 2017). The safety and efficacy of pembrolizumab as a 1L treatment was tested in cohorts 2 and 3, in combination with standard chemotherapy (cohort 2), and a step further in cohort 3, as a monotherapy (Table 1; Bang et al., 2017a; Catenacci Daniel et al., 2017). The preliminary results from cohort 1, comprising 259 patients, were presented at the American Society of Clinical Oncology (ASCO) meeting in 2017. Pembrolizumab demonstrated promising efficacy as 3L treatment in heavily pretreated patients with advanced-stage GC. Survival and biomarker data, including MSI statuses from KEYNOTE 059, are pending **[NCT 02335411]**. Based on these results, FDA approved pembrolizumab for the treatment of patients with PD-L1-positive recurrent or advanced-stage GC/GEC, who have received two or more lines of chemotherapy, including fluoropyrimidine- and platinum-based chemotherapy, and HER2-neu-targeted therapy in eligible patients. Similarly, results from cohort 3 supported the feasibility of single-agent pembrolizumab as 1L treatment in advanced-stage GC/GEC.

Following these promising results, two phase III trials are currently underway to test pembrolizumab as 1L and 2L treatments for patients with advanced-stage GC. KEYNOTE 61 is a phase III, open-label, randomized controlled trial (RCT) of pembrolizumab vs. paclitaxel in subjects with advanced-stage GC who progressed after 1L platinum and fluoropyrimidine treatment (31)(31)(31). In this trial, 720 patients are to be enrolled to receive either pembrolizumab (200 mg IV on Day 1 of each 21-d cycle) or paclitaxel (80 mg IV on Days 1, 8, and 15, over a 28-days cycle). PFS and OS in PD-L1-positive patients are the primary endpoints^3^. A preliminary analysis suggests that pembrolizumab has missed the primary endpoint. The hazard ratio (HR) for OS was 0.82 (0.66–1.03)^4^. This drug also failed to show improvement in PFS according to data released by the trial sponsors. The complete results from the trial are awaited **[NCT 02370498]**. KEYNOTE 62 is a randomized, active-controlled, biomarker-selected phase III trial to evaluate the survival benefit of pembrolizumab as a 1L single agent or in combination with standard chemotherapy, compared with standard chemotherapy alone, in patients with advanced-stage GC^5^. In this trial, 750 patients are projected to be enrolled and randomized in either the pembrolizumab arm, pembrolizumab plus cisplatin and 5-fluorouracil (FU), or placebo plus cisplatin and 5-FU arm. This trial is designed to investigate the superiority of pembrolizumab and chemotherapy, in combination, over chemotherapy alone and non-inferiority of single-agent pembrolizumab compared with that of standard chemotherapy. PFS is the primary endpoint, with secondary endpoints of OS and ORR **[NCT 02494583]**.

Another PD-1 inhibitor, nivolumab, was tested in ONO 4538-12, a phase III, double-blind, placebo-controlled trial in Japanese patients (Kang et al., 2017b). Patients with advanced-stage/recurrent GC/GEC irrespective of PD-L1 expression were randomized to receive nivolumab and placebo in a 1:1 proportion. PD-L1 expression was examined only retrospectively using IHC. The primary endpoint was OS. Approximately 75% of patients had metastases to more than two organs and 80% of patients had received at least three lines of chemotherapy in this group. Complete remission was not achieved; however, partial remission was observed in 11% of these heavily treated patients compared with that achieved with placebo. This is extremely promising as there is no alternative recommended therapy for patients who have suffered progression after multiple lines of chemotherapy. Although in absolute terms, the gain in OS for nivolumab was only 1.1 months, it reduced the mortality risk by 37% compared with that achieved using the placebo. Moreover, the survival benefit with nivolumab persisted for more than 12 months and survival curves did not cross; these findings were contrary to those of most other trials of chemotherapy or targeted therapy in 2L and 3L settings, where therapeutic benefit decreased with time (Kang et al., 2012; Ford et al., 2014; Li et al., 2016). The survival advantage was independent of PD-L1 status. Of note, the PD-L1 positivity rate was only 13% patients in this study. The HR of nivolumab were less than 1 in most patient subgroups, including by age, male gender, histology, the absence of liver and peritoneal metastases, and metastases in more than two organs. The HR in this study were better than for previous agents tested in previous studies in this setting; however, such a comparison is limited by different patient characteristics at baseline (Catalano et al., 2009). Although nivolumab was well tolerated in these patients with the rate of adverse effects similar to those in other trials, quality of life data were not reported in this study. Further studies should be performed to address this limitation **[NCT 02267343]**.

##### PD-l1 inhibitors (avelumab and durvalumab)

Avelumab is a fully humanized immunoglobulin G1 (IgG1) anti-PD-L1 antibody that inhibits the PD-1/PD-L1 interaction but does not impair the PD-1/PD-L2 pathway (Grenga et al., 2016). It also affects antibody-dependent cell-mediated cytotoxicity (ADCC), as demonstrated in preclinical experiments (Boyerinas et al., 2015). The safety and clinical activity of avelumab in GC were studied in a cohort of 20 Japanese patients with PD-L1-unselected advanced-stage GC in a phase Ib dose expansion study (Nishina et al., 2016). The study suggested that the safety and clinical activity profiles for the treatment of advanced-stage GC with the single-agent avelumab were favorable, although PD-L1-positive patients tended to respond better than PD-L1-negative patients **[NCT 01943461]**.

The safety and clinical activity of avelumab as 2L and first-line maintenance (Mn) therapy was studied in 99 PD-L1 unselected and 62 patients with advanced-stage GC/GEC, respectively Table 1 (Chung et al., 2016). In the Mn group, ORR and PFS were higher in PDL-1-positive patients than in PD-L1-negative patients; in the 2L group, ORR was higher in PD-L1-positive patients whereas the PFS was lower **[NCT 01772004]**.

Two phase III RCTs of avelumab are ongoing: JAVELIN 300 and JAVELIN 100 (Moehler et al., 2016, 2018; Eric et al., 2017). Subjects are currently being recruited for JAVELIN 300, which aims to investigate the utility of avelumab as a 3L agent in comparison with the best supportive care for patients with recurrent, locally advanced, or metastatic GC/GEC **[NCT 02625610]**. JAVELIN 100 aims to compare single-agent avelumab (10 mg/kg every 2 weeks) therapy with the continuation of 1L chemotherapy (5-FU/leucovorin or capecitabine plus oxaliplatin) as Mn therapy **[NCT 02625623]**.

Another PD-L1 inhibitor, durvalumab, is a highly selective humanized monoclonal antibody that blocks the binding of PD-L1 to CD80 and PD-1. As a single agent, this compound achieved an acceptable safety profile in heavily pretreated patients with GC/GEC, when dosed at 10 mg/kg every 2 weeks (Segal et al., 2014) **[NCT 01693562]**. A phase Ib/II study of durvalumab as 2L or 3L treatment in advanced disease is currently underway in patients with GC/GEC to test the role of durvalumab and tremelimumab as single-agent and combination therapy (Kelly et al., 2015) **[NCT 02340975]**.

##### Adjuvant and combination therapies

The role of ICIs as adjuvant or neoadjuvant therapy in stage 2 or 3 GC is being explored in the phase III CheckMate-577 trial (Kelly et al., 2017). Approximately 760 patients with resected GC/GEC will be randomized to receive placebo or nivolumab (240 mg every 2 weeks) for 16 weeks, followed by nivolumab (480 mg every 4 weeks) for 1 year. The primary endpoints are OS and disease-free survival (DFS). It has been shown that following neoadjuvant therapy, chemoradiation induces an ICI-favorable microenvironment characterized by an increase in tumor lymphocytes and lymphocyte clusters in perivascular spaces (Thompson et al., 2016). The use of ICIs for adjuvant therapy is appealing as the administration of ICIs before the development of metastases may induce immune-mediated cancer regression (Kelly, 2017) **[NCT 02743494]**.

Akin to the treatment approach for other tumors, the safety and efficacy of a combination of chemotherapy and immunotherapy was tested in patients with GC. Pembrolizumab, in combination with standard chemotherapy, was tested as 1L treatment for patients with advanced-stage GC in cohort 2 of the KEYNOTE-059 study ((Bang et al., 2017a); Table 1). Pembrolizumab in combination with 1L chemotherapy (5-FU plus cisplatin) promoted antitumor activity and showed a favorable safety profile as 1L therapy for patients with advanced GC/GEC. This modality warrants further testing **[NCT 02335411]**.

The efficacy and safety of nivolumab alone and in combination with ipilimumab (a CTLA-4 inhibitor) was tested in the phase I/II CheckMate 032 Table 1 trial (Janjigian et al., 2017): 160 pretreated patients with GC/GEC (of whom 79% were treated with at least two lines of chemotherapy) were enrolled into three groups. Nivolumab as monotherapy, or in combination with ipilimumab, elicited a strong response and good OS in heavily pretreated western patients with advanced GC/GEC. Although the data suggested benefits of a combination of ipilimumab and nivolumab in comparison with the use of either agent alone, patients in the combination therapy arm also suffered an increased number of adverse events. Despite this, the safety data were consistent with prior reports (Kumar et al., 2017) **[NCT 01928394]**.

ICIs are also being tested in combination with several novel agents and external beam radiotherapy for the treatment of advanced-stage GC/GEC (Table 2). A phase II trial comparing BMS-986016, an anti-LAG-3 monoclonal antibody, in combination with nivolumab, with the nivolumab plus ipilimumab combination is underway **[NCT 02935634]**. LAG-3 resembles CD4 and is encoded by the *LAG-3* gene, which belongs to the immunoglobulin superfamily (IgSF) (He et al., 2016). LAG-3 is expressed by the natural killer cells (NK), B-cells, TIL, and DCs. It competes with CD4 to bind the major histocompatibility complex (MHC) II. The binding of LAG-3 to MHC II molecules results in the down-regulation of CD4^+^ antigen-specific T-cell proliferation and cytokine secretion. LAG-3 is expressed on both Tregs and anergic T-cells (He et al., 2016).

**Table 2:** Table showing ongoing trials on emerging therapies in gastric cancer.

**Clinical Trial.gov identifier**	**Intervention used**	**Estimated sample size^#^**	**Phase**	**Primary endpoints**	**Current status (as on 08/2017)**
**CHECKPOINT INHIBITORS AS MONOTHERAPY OR IN COMBINATION**					
NCT02935634	Nivolumab+ BMS-986016 vs. Nivolumab+ Ipilimumab	300	Phase II	ORR DOR PFS	Recruiting
NCT02488759	Nivolumab mono or (N+ Ipilimumab/BMS-986016/Daratumumab)	500	Mono- Phase I Combination- Phase II	ORR, Incidence of AEs, Rate of surgery delay	Recruiting
NCT01928394	Nivolumab mono or N+ Ipilimumab or N+ Ipilimumab+ Cobimetinib	1150	Phase I Phase II	ORR	Recruiting
NCT02864381	Nivolumab mono or N+ Andecaliximab	144	Phase II	ORR	Active, not recruiting
NCT02830594	Pembrolizumab+ EBRT	14	Phase II	Comparison of molecular biomarker and disease outcome	Recruiting
NCT02730546	Pembrolizumab+ RT+ Surgery+ CT (Carboplatin+ Fluorouracil + Leucovorin + Oxaliplatin)	68	Phase I Phase II	Path CR (Complete response), PFS	Recruiting
NCT02443324	Pembrolizumab+ Ramucirumab	155	Phase I	Dose limiting toxicities	Recruiting
NCT02393248	Pembrolizumab+ INCB054828 Or Gemcitabine+ Cisplatin+ INCB054828 Or Docetaxel+ INCB054828 Or Trastuzumab+ INCB054828	280	Phase I Phase II	Maximum tolerated dose as mono (INC) or in combination	Recruiting
NCT02335411	Pembrolizumab mono (treatment naïve) OR Pembrolizumab mono (previously treated) OR P+ Cisplatin+ 5-FU+ Capecitabine (Treatment naïve)	253	Phase II	Adverse events, Discontinuing study due to AE, ORR	Active, Not recruiting
NCT02318901	Pembrolizumab Mono OR P+ Ado-trastuzumab etamine OR+ P+ Cetuximab	90	Phase I Phase II	Recommended phase 2 dose of trastuzumab with pembrolizumab	Active, Not recruiting
NCT02658214	Durvalumab+ Oxaliplatin+ 5-FU+ Leucovorin (Cohort 5 for GC/GEC)	60	Phase I	Safety/tolerability of first line therapy Incidence of adverse events	Recruiting
NCT02625623 [JAVELIN 300]	Avelumab mono with best supportive care OR Physician choice CT (Irinotecan OR Paclitaxel)+ BSC OR BSC alone	376	Phase III	OS	Active, Not recruiting
NCT02625610 [JAVELIN 100]	Induction with Oxaliplatin and 5-FU OR Capecitabine f/b Maintenance with Avelumab OR Induction with Oxaliplatin and 5-FU OR Capecitabine and Maintenance with either continuation of induction CT or avelumab only if disease progression/deterioration clinically/unacceptable toxicity/or discontinuation	466	Phase III	OS, PFS	Recruiting
NCT01772004	Avelumab for GC/GEC (first line switch maintenance and second line): Among 8 secondary cohort GC/GEC (3L) Among 4 Efficacy expansion cohorts	1756	Phase I	Dose limiting toxicity and best overall response	Recruiting
NCT02746796	ONO-4538+ SOX (Part 1) ONO-4538+ Cape OX (Part 1) ONO-4538+ Chemo group (Part 2) → either SOX or Cape OX Placebo+ Chemo group (Part 2)	680	Phase II Phase III	PFS OS	Recruiting
NCT02678182	No intervention **(A1)** Capecitabine maintenance **(A2)** MEDI4736 mono **(A3)** Trastuzumab Maintenance **(B1)**	616	Phase II	PFS	Recruiting
NCT02572687	MEDI4736+ Ramucirumab	114	Phase I	Dose limiting toxicity	Recruiting
NCT02563548	PEGPEM (PEGPH20+ Pembrolizumab)	81	Phase I	Efficacy of combination of PEGPH20 and pembrolizumab	Recruiting
NCT02393248	**Dose expansion:** INCB054828+ Gemcitabine+ Cisplatin OR Pembrolizumab+ INCB054828 OR Docetaxel+ INCB054828 OR Trastuzumab+ INCB054828	280	Phase I Phase II	Maximum tolerated dose and Pharmacodynamics of INCB054828	Recruiting
NCT02318277	Combination of MEDI 4736+ INCB024360	185	Phase I	Dose limiting toxicities, adverse events, ORR	Recruiting
NCT02268825	MK-3475 (pembrolizumab) in combination with mFOLFOX6	39	Phase I	Safety of combination of FOLFOX and MK-3475	Active, Not recruiting
NCT02903914	INCB001158 (CB-1158) alone or in combination with Pembrolizumab	346	Phase I Phase II	Safety, pharmacokinetics, biomarkers and tumor response.	Recruiting
**NAPABUCASIN (FORMERLY BBI608)**					
NCT02178956	BB1608+ Paclitaxel vs. Placebo+ Paclitaxel	700	Phase III	OS	Active, Not recruiting
NCT02024607	**A**:BBI608+FOLFOX6 **B**:BBI608+FOLFOX6+ Bevacizumab **C**:BBI608+ CAPOX **D**:BBI608+ FOLFIRI **E**:BBI608+ FOLFIRI+ Bevacizumab **F**:BBI608+ Regorafenib **G**:BBI608+ Irinotecan	609	Phase I Phase II	Adverse events, ORR	Recruiting
**ANDECALIXIMAB (FORMERLY GS 5745)**					
NCT02545504	Andecaliximab+ mFOLFOX6 (Leucovorin+ 5-FU+ Oxaliplatin) Placebo+ mFOLFOX6	432	Phase III	OS	Active, not recruiting
NCT01803282	Andecaliximab mono OR Andecaliximab+ Chemotherapy (mFOLFOX6)	261	Phase I	Incidence of adverse events (safety)	Recruiting
NCT02862535	Andecaliximab mono OR Andecaliximab+ S-1+ Cisplatin	18	Phase I	Overall safety	Active, not recruiting
**OLAPARIB (PARP INHIBITOR)**					
NCT02734004	Olaparib+ MEDI 4736	147	Phase I Phase II	Disease control rate Safety and tolerability	Active, not recruiting

^*^ORR, Overall response rate; DOR,: duration of response; OS, overall survival. PFS progression # many phase 1 and 2 trials are basket trials and sample sizes may be for multiple tumor types. BMS-986016 anti-LAG 3 inhibitor; INCB054828 Pan FGFR tyrosine kinase inhibitor; $Daratumumab CD38 inhibitor; MEDI 4736 Durvalumab; MK 3475 Pembrolizumab; INCB024360 (also Epacadostat) IDO-1 inhibitor; Cobimetinib mitogen activated protein kinase 1/2 inhibitor; INCB001158 (CB-1158) Arginase Inhibitor.

LAG-3 acts in a manner similar to CTLA-4 and exhibits synergistic activity with PD-1/PD-L1. PD-1 and LAG-3 signaling inhibits CD8 activity via antigen and cytokine signaling (Woo et al., 2012). A negative regulation of CD8-positive TILs by LAG-3 and PD-1 has been demonstrated in the murine models of ovarian cancer. The levels of two pro-inflammatory cytokines, interferon gamma (IFN-γ) and tumor necrosis factor alpha (TNF-α), are significantly reduced in tumor microenvironments containing CD8^+^LAG-3^+^PD-1^+^ T-cells compared with those in the tumor milieu containing LAG-3^+^ or PD-1^+^CD8^+^ T-cells (Huang et al., 2015). In addition, LAG-3 acts synergistically with PD-1 to diminish the antitumor response by regulating T-cell function (Grosso et al., 2009). The expression of PD-1 and LAG-3 has been shown to be up-regulated following the development of GC, which suggests that immunotherapy targeting PD-1 and LAG-3 represents a possible strategy for the treatment of GC (Takaya et al., 2015).

Other promising agents for combination therapy include PEGylated recombinant human hyaluronidase (PEGPH20), which is developed using the Halozyme's proprietary local dispersion of co-administered drugs. Recombinant human PH20 (rHUPH20) is a hyaluronidase with an activity similar to hyaluronidase-5 (Kultti et al., 2012). The clinical utility of rHUPH20 is limited by the short circulation half-life (2.3 min) of its PEGylated form, whereas PEGPH20 has a long half-life of 10.3 h in circulation (Thompson et al., 2010). Hyaluronic acid (HA) is a large, unbranched, non-sulfated glycosaminoglycan. It is integral to the extracellular matrix (ECM). In several malignancies, including the pancreatic, lung, breast, gastric, colon, and prostate cancer, large intact HA molecules are generated in the tumor microenvironment because of the elevated activity of HA synthase. These molecules are either rapidly incorporated into the ECM that surrounds the tumor cells, or remain at the cell surface, engaged by HA-binding receptors, interacting glycoproteins, and proteoglycans (Kultti et al., 2012). The ability of HA to absorb water in the ECM, along with other matrix components, leads to the swelling of tissue spaces, resulting in the high interstitial fluid pressures observed in tumor foci. The interstitial fluid pressure of the tumor microenvironment may be elevated up to 30 times, leading to a compression of the blood vessels, hypoxia, and drug resistance. HA also forms a protective shield around the tumor cells, which inhibits the translocation of drug molecules to the tumor foci (Janet et al., 2012; Kultti et al., 2012). In a study of 215 patients with GC, up to 40% of the tumors exhibited an HA labeling index of 30–40% (Setälä et al., 1999). HA-positive tumors were poorly differentiated, more invasive, at a more advanced stage, and associated with worse outcomes upon univariate analysis than HA-negative tumors (Setälä et al., 1999). PEGPH20 breaks-down HA to facilitate entry of co-administered anticancer therapeutic agents and activate immune cells in tumor microenvironments. In addition, the removal of HA from the tumor milieu helps to restore previously constricted vessels, which increases the access of immune cells to tumor microenvironments (Kultti et al., 2012) **[NCT 02563548]**.

Phase I/II trials of other molecules in combination with ICIs include those investigating INCB054828, a pan-inhibitor of FGFR types 1, 2, and 3; cobimetinib, a mitogen-activated protein kinase (MEK) inhibitor approved for use in combination with vemurafenib for advanced melanoma patients with recurrent *BRAF* V600E or *BRAF* V600K mutations; daratumumab, an anti-CD38 monoclonal antibody approved for treatment of patients with multiple myeloma; **INCB024360 (also Epacadostat), an IDO-1 inhibitor; and INCB001158 (CB-1158) an arginase Inhibitor [NCT 02393248, NCT 01928394, NCT 02488759, NCT02318277, NCT02903914]**. The ongoing trials of these novel therapies have been compiled in Table 2.

##### Other immunotherapy

Several forms of immunotherapy, including adoptive T-cell therapies that have shown efficacy against other tumor types, are undergoing clinical trials involving patients with advanced-stage GC/GEC (Table 3). A novel form of immunotherapy, chimeric antigen receptor T-cell (CAR-T) therapy that has been approved for the treatment of some hematological malignancies, needs special mention here because it may also be a promising therapy for patients with advanced-stage GC/GEC in the future (Zhang et al., 2016). Briefly, CARs are genetically modified receptors that identify tumor Ag in a non-MHC-restricted fashion, contrary to normal T-cells whose activity against tumor cells is limited by MHC (Dai et al., 2016). CAR consists of a single-chain fragment variable (scFv), which is formed by heavy and light chain amino acid sequences connected by a short linker, a hinge region, and a transmembrane component that connects it to an intracellular domain. The intracellular domain is the CD3ζ immuno-receptor tyrosine-based activation motif (ITAM) domain, which activates T-cells. The scFv engages the target, which triggers downstream signaling, thereby activating T-cells independent of MHCs. CAR-T cell therapy has evolved over time. Depending upon the type of co-stimulatory molecules, this form of therapy has been classified as first to fourth generation. Fourth-generation CAR-T cells secrete cytokines, such as interleukin (IL) 12, that regulate the tumor microenvironment; these cells are also known as TRUCK cells (Chmielewski et al., 2014). The major challenges in the application of this therapy to solid tumors include the identification of tumor antigens that act as targets for CAR-T therapy.

**Table 3:** Other promising immunotherapies in patients with advanced stage gastric carcinoma.

**Therapy**	**Mechanism in gastric cancer**	**Clinical trial number status**
INCAGN01876 + Pembrolizumab + Epacadostat (previously INCB24360)	**INCAGN01876**: An anti-human glucocorticoid-induced tumor necrosis factor receptor (TNF 18; GITR; CD357) agonistic humanized monoclonal antibody, with immune checkpoint modulating activity. Anti-GITR antibody INCAGN01876 binds to and activates GITRs present on T-cells activating tumor-antigen-specific T-effector cells (T effs), and suppressing the function of activated T-regulatory cells (Tregs). (Knee et al., 2016) **Epacadostat:** An inhibitor of IDO-3, which is an immunosuppressive enzyme that acts as an immune checkpoint.(Jochems et al., 2016)	NCT03277352 l Recruiting l Phase I l Phase II
l INCAGN01949 +Nivolumab l INCAGN01949 + Ipilimumab l INCAGN01949 + Ipilimumab + Nivolumab	**INCAGN01949** is a humanized IgG1 agonistic monoclonal antibody which acts on a T cell co-stimulatory receptor, OX40 (CD134, TNFRSF4). This potentiates signaling by T cell receptor (TCR) during priming of CD4+ and CD8+ T cells and memory T cell recall responses. Besides, it also co-engages Fcγ receptors expressed by tumor-associated T effs which facilitates the selective depletion of intratumoral Tregs (Gonzalez et al., 2016).	NCT03241173 l Recruiting l Phase I l Phase II
Gene therapy using anti-KRAS G12V mTCR cells.	HLA-A1101-restricted murine T-cell receptors (mTCR) are generated that specifically recognize the G12V-mutated variant of KRAS (and other RAS family genes). A retrovirus vector is used to transduce alpha and beta chains of these receptors into the peripheral blood lymphocytes. mTCR transduced T cells recognize and lyse HLA-A1101+ target cells, expressing this mutated oncogene (Wang et al., 2015).	l NCT03190941 l Recruiting l Phase I l Phase II
CRS-207 in combination with pembrolizumab	**CRS-207** is a live, attenuated, double-deleted (LADD) Listeria monocytogenes. It induces both, an innate as well as a T cell-mediated immunity. A modified form of CRS-207 expresses a tumor associated antigen mesothelin, which is expressed in GC. (Nemunaitis et al., 2008). However, CRS-207 development program has been stopped recently after preliminary results (Biotech, 2018).	NCT03122548 l Active not recruiting l Phase II
CBT-501 (Genolimzumab)	Novel PD-1 inhibitor (Bang et al., 2017b)	NCT03053466 l Recruiting l Phase I
MCLA-128 (bispecific)	IgG1 Bispecific Antibody against HER2 and HER3 (Calvo et al., 2016).	NCT02912949 l Recruiting l Phase I l Phase II
AbGn-107	An antibody drug conjugate (ADC) against antigen AG7, present on GC/GEC cells (NCI Drug DictionaryDictionary, 2018a).	NCT02908451 l Recruiting l Phase I
Adoptive T-cell therapy with HER2Bi armed T-cells	These are activated T cells (ATC) coated with anti-CD3 × anti-Her2 bispecific antibodies (Her2Bi) which have antineoplastic and immunomodulating properties. These activated T-cells attach *in vivo* to the T-cells (CD3-expressing) and the tumor cells (HER2/neu +) thereby cross-linking the T cells with tumor cells. This leads to enhanced immunological response characterized by the recruitment and activation of cytotoxic T lymphocytes (CTLs) and secretion of antitumor cytokines (Yu et al., 2017).	NCT02662348 l Unknown l Phase I
Adoptive T cell therapy using Tumor infiltrating lymphocytes (TILs)	Immunotherapy Using Tumor Infiltrating Lymphocytes (Rosenberg, 2018).	NCT01174121 l Recruiting l Phase II
Adoptive T cell therapy with Infusion of iNKT cells and CD8+T cells	Invariant Natural killer T (iNKT) cells recognize KRN7000 which is up-regulated in many cancers. NKT cells directly lyse the CD1d expressing tumor cells by using perforins. The infusion of iNKT cells and PD-1+CD8+T cells may reduce the tumor burden and improve survival (Wolf et al., 2018).	NCT03093688 l Recruiting l Phase I l Phase II

In GC, CAR-T therapy against four major antigens is currently trialed. First, *HER2* gene amplification and overexpression of its product (p185) has been reported in up to one-third of gastric tumors. The overexpression of this protein does not occur in normal gastric cells, making it an ideal target for therapy. A trial that aims to study the adverse effects of anti-HER2 CAR-T cell therapy in patients with advanced-stage HER2-positive GC/GEC is underway **[NCT02713984]**. Next antigen, carcinoembryonic antigen (CEA) is overexpressed in gastric, pancreatic, colorectal, and hepatocellular carcinoma; its overexpression portends poor prognosis in GC (Deng et al., 2015). Patients are currently being enrolled for a trial investigating the utility of anti-CEA CAR-T cell therapy in advanced-stage CEA-positive GC **[NCT02349724]**. Another protein, mucin 1 is encoded by *MUC1*, with differential glycosylation activities in normal and tumor cells. Anti-MUC1 CAR-T cells against MUC1 antigen showing abnormal glycosylation patterns are also being investigated in patients with advanced-stage MUC 1-positive GC/GEC **[NCT02617134]**. Finally, CAR-T cell therapy against epithelial cell adhesion molecule (EpCAM) is evaluated. EpCAM is a stem cell marker associated with aggressive gastric carcinoma. Anti-EpCAM CAR-T cell therapy is under trial for patients with advanced-stage EpCAM-positive GC/GEC **[NCT03013712]**. The mentioned trials are all phase I/II trials, designed primarily to evaluate the toxicity profile of CAR-T cells in GC/GEC. These trials are currently recruiting patients; data on the survival time of CAR-T cells in patients with advanced-stage GC/GEC and anti-tumor efficacy will also be collected. In addition to the identification of target antigens, the cost and management of adverse effects represents a challenge (Hartmann et al., 2017). Major adverse effects include on-target off-tumor toxicities similar to those observed in autoimmune diseases. These result from the sharing of antigens between tumor and healthy cells. Neurotoxicity (linked to the release of IL-2) and cytokine release syndrome (linked to the release of IL-6, IFNγ, and TNF-α) are potentially fatal if not diagnosed in a timely manner. The development of dual CAR-T cells and antigen-specific inhibitory CAR (iCAR) would increase the selectivity, and thereby safety, of this therapy (Wang et al., 2017).

##### IMU-131 HER2/neu peptide vaccine

IMU-131, a novel target for vaccines for patients with HER 2-positive GC/GEC, is a single peptide composed of three individual B-cell epitope peptides, P4, P6, and P7, which are the components of the HER2/neu structure^6^. Antibodies against IMU-131 bind these three separate regions of the HER2 receptors and dimerization loop, thereby preventing dimerization and inhibiting intracellular signaling. Such blockade of the HER2 signaling pathway is considered more robust than blockade using trastuzumab alone. An earlier version of this vaccine demonstrated safety and immunogenicity, but was limited by lack of stability (Clinicaltrial.Gov, 2018). The new vaccine is considered relatively more immunogenic and stable [NCI Drug (Dictionary, 2018b)]. A phase Ib/2 open label trial with 18 patients is currently underway. The trial will evaluate the safety and tolerability of the IMU-131 HER2/neu peptide vaccine, and also identify the recommended phase 2 dose in combination with standard chemotherapy (cisplatin and 5-FU or capecitabine) in patients with HER2/neu-overexpressing advanced GC/GEC [***NCT02795988***].

### Anti-claudin 18.2 (CLDN 18.2) antibody (claudiximab)

CLDN 18.2 is an isoform of claudin proteins, which are structural components of tight junctions present in the paracellular region. They are required for barrier maintenance, paracellular transport, and signal transduction (Sahin et al., 2008; Caron et al., 2015). Their expression in the normal gastric mucosa is exclusively limited to differentiated epithelial cells that are absent in the gastric stem cell zone and preserved in malignant transformations in a considerable number of patients with primary and metastatic GC (Sahin et al., 2008). CLDN 18.2 is a CD20-like differentiation protein that is overexpressed in non-small cell lung cancers (NSCLCs; 25%), gastric (70%), pancreatic (50%), and esophageal (30%) cancers (Sahin et al., 2008)^7^. CLDN 18.2 expression is apparent in up to 77% of patients with GC as confirmed by membrane staining methods. Further, CLDN 18.2 protein expression was observed in 56% of patients with GC, as confirmed by CLDN 18.2 expression of ≥2+ in at least 60% of cells. The expression of this protein is affected by the ethnic background, with higher expression levels observed in Japanese patients than in Caucasian patients (Matsuda et al., 2007; Sahin et al., 2008).

**Figure 3:**
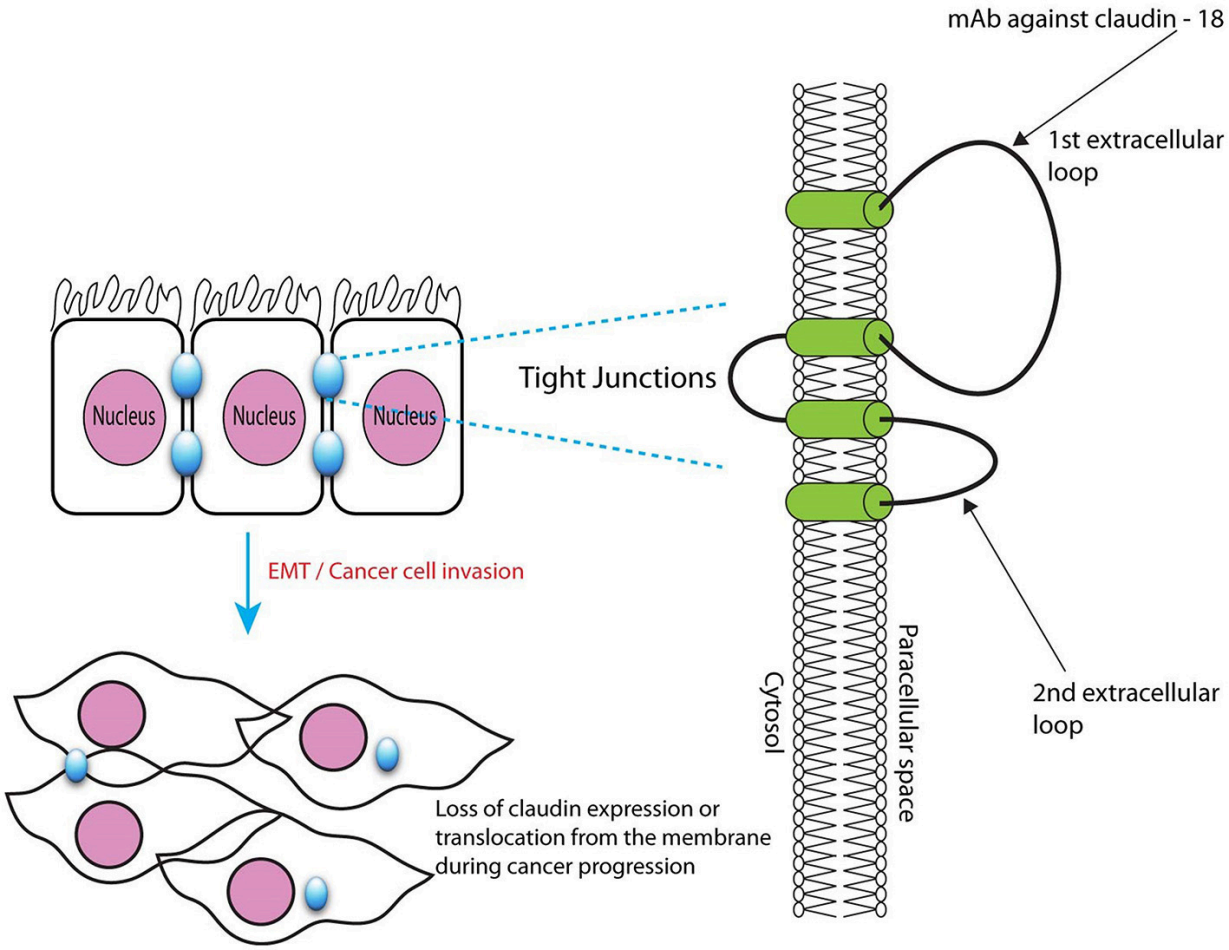
Microscopic structure of tight junctions showing claudin 18.2 with binding site for monoclonal antibody claudiximab.

The novel chimeric idealized monoclonal antibody (IMAB) 362, now known as claudiximab, is a first-in-class agent that targets only tumor cells and exhibits a lower toxicity profile than other antineoplastic monoclonal antibodies (Claudiximab, 2017; Figure 3). This IgG1 antibody activates ADCC and complement-dependent cytotoxicity (CDC). When combined with chemotherapy, it acts as an immunomodulatory agent and facilitates T-cell infiltration into the tumor microenvironment. It is being tested both as monotherapy and in combination with chemotherapy for the treatment of NSCLC, and gastric, esophageal, and pancreatic cancers (Claudiximab, 2017).

The safety and efficacy of repeated doses of claudiximab (300 and 600 mg/m^2^) monotherapy in patients with refractory/metastatic CLDN 18.2-positive GC/GEC was studied in a phase IIa MONO trial that included 54 patients Table 1 (Trarbach et al., 2014). Four patients received claudiximab at a dose of 300 mg/m^2^, while 50 patients received a higher dose of 600 mg/m^2^. The PFS was 102 days with a DCR of 30%. The trial suggested the safety and feasibility of claudiximab at a dose of 600 mg/m^2^ in patients with advanced-stage GC/GEC. **[NCT 01197885]**. However, in subsequent trials of claudiximab, higher doses (800 mg/m^2^ and 1,000 mg/m^2^) were used and found to be safe.

The safety and efficacy of claudiximab in GC were assessed in a phase IIb trial Table 1 (Schuler et al., 2016). This Phase II First-Line Therapy in Patients with Advanced CLDN18.2^+^ Gastric and GEJ Adenocarcinoma (FAST) trial tested the benefit of adding claudiximab to 1L chemotherapy in inoperable or recurrent GC and GEC. Eligible patients showed a CLDN 18.2 expression level of ≥2+ in ≥40% tumor cells, as demonstrated by IHC-based (CLAUDETECT) testing. Claudiximab with EOX significantly improved PFS, OS, and ORR. The response correlated with the level of CLDN 18.2 expression and was greater in patients with 70% or more CLDN 18.2-expressing tumor cells. In the FAST trial, claudiximab was well tolerated with a slight, although not statistically significant, increase in adverse events compared with chemotherapy. Vomiting and neutropenia were slightly more frequent with claudiximab. Another important finding that was recently highlighted was the occurrence of CLDN 18.2 expression in patients who were mostly negative for the actionable target HER2-neu. Of 154 patients with known mutation status for both HER2-neu and CLDN 18.2, 94 were CLDN 18.2-positive. Among these 94 patients, only 13 (14%) expressed HER2-neu. Therefore, CLDN 18.2 may represent a useful non–HER2-neu overlapping target (Schuler et al., 2017) **[NCT 01630083]**.

Preliminary results from the concomitant phase I PILOT trial, which aims to evaluate the safety and efficacy of claudiximab combined with zoledronic acid (ZA) and IL-2 in patients with CLDN 18.2-positive GEC, also indicate the efficacy of claudiximab against GC (Sahin et al., 2015; Table 1). Although primary endpoints (which include biomarkers to assess the immunomodulatory effect of claudiximab) have not yet been reported, the safety profile and clinical outcomes are promising. These results suggest that claudiximab possesses a single-agent antitumor activity and that the safety profile is not compromised when combined with ZA/IL-2 for the treatment of advanced GECs. Analysis of data for primary endpoints (biomarkers) should further establish whether the ZA/IL2 combination augments the immunomodulatory effects of claudiximab **[NCT 01671774]**.

Finally, results from the FAST trial have paved the way for an imminent confirmatory phase III trial to be carried out by Ganymed (Claudiximab, 2017; Newswire, 2017)^7^. Furthermore, claudiximab has been granted orphan drug status by the European Union (2010) and FDA (2012) for the treatment of advanced, unresectable GC (Claudiximab, 2017).

### Matrix metalloproteinase-9 (MMP-9) inhibitor (andecaliximab)

MMP-9 is a gelatinase that directly degrades ECM, thereby modulating the tumor microenvironment (Farina and Mackay, 2014). It is required for remodeling of the ECM, including collagen IV and laminin in the basement membrane, and facilitation of tumor invasion and metastases. MMP-9 activates latent cytokine and growth factors, including TNF-α, tumor growth factor beta (TGF-β), IL-1β, and IL-8, and alters the expression of cell-surface proteins in lymphoid and myeloid cells (Farina and Mackay, 2014). Its overexpression is correlated with poor prognosis of GC. In a meta-analysis by Zhang et al. (2012), comprising 1700 patients from 11 studies, positive MMP-9 expression had an adverse impact on OS in patients with GC (HR 1.25, 1.11–1.40) (Zhang et al., 2012). Chen et al. (2015) reported higher expression of MMP-9 in the GC tissue (86.67%) than in the adjacent healthy tissue (10.00%). These authors additionally identified an association between MMP-9 overexpression and tumor depth. Namely, the preoperative serum levels of MMP-9 correlated with the advanced stage and the presence of lymph node metastases (Chen et al., 2015).

**Figure 4:**
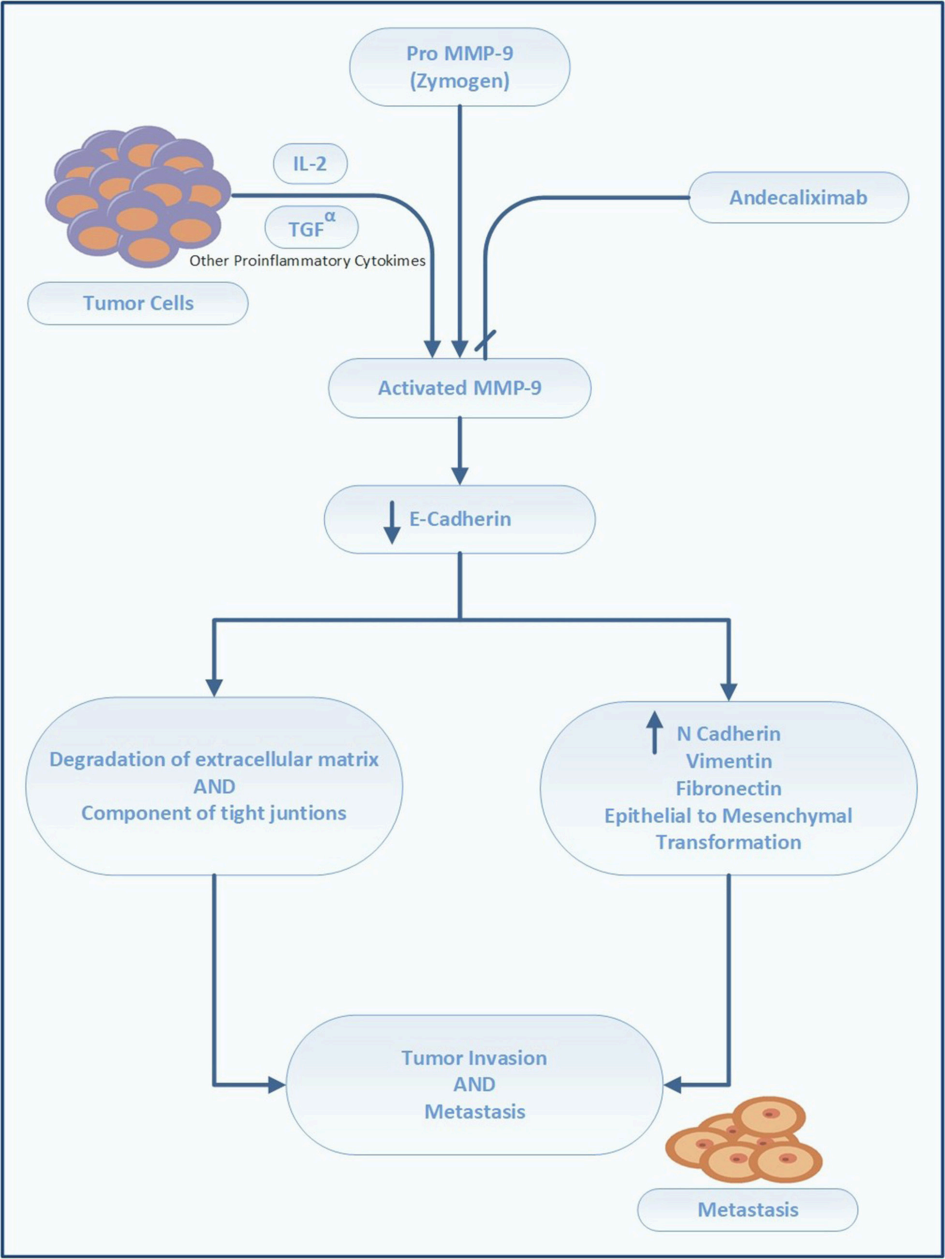
Mechanism and site of action of andecaliximab.

The monoclonal antibody andecaliximab (formerly GS 5745) is an inhibitor of human MMP-9. It binds MMP-9 at the junction between the propeptide and catalytic domains, distal to the active site, and acts by preventing the activation of inactive zymogen (Figure 4). In addition, this antibody acts as an allosteric inhibitor (Marshall et al., 2015). Andecaliximab is under development for the treatment of cystic fibrosis, solid tumors (gastric, pancreatic, and NSCLC), and inflammatory disorders (rheumatoid arthritis, Crohn's disease, and ulcerative colitis)^8^.

The safety and activity of andecaliximab were tested in a phase I trial in patients with advanced GC/GEC Table 1 (Bendell et al., 2017b). Forty patients with HER2-neu-negative advanced or metastatic GC received modified folinic acid, fluorouracil, and oxaliplatin (mFOLFOX), and andecaliximab (800 mg IV) every 2 weeks. The combination of FOLFOX with andecaliximab was well tolerated and demonstrated therapeutic activity, particularly in treatment-naive patients **[NCT 01803282]**.

Patients with advanced GC/GEC in the U.S., Australia, Poland, Hungary, Spain, and Turkey are currently being recruited for a phase III randomized, double-blind, placebo-controlled trial (Bendell et al., 2017a) that aims to compare the efficacy of andecaliximab vs. placebo in combination with mFOLFOX. The planned primary outcome is OS, and the secondary outcomes are PFS and ORR. The targeted enrollment is set at 432 patients, and the treatment to be administered is andecaliximab (800 mg, IV) on Days 1 and 15 of each 4-week cycle, with FOLFOX according to the standard of care. The control arm includes treatment-naive patients who will receive a placebo plus FOLFOX **[NCT 02545504]**.

Another phase II trial is planned to evaluate the safety and efficacy of the combination of nivolumab (3 mg/kg) and andecaliximab (800 mg, IV) vs. nivolumab (3 mg/kg alone) for recurrent or advanced GC; this trial aims to enroll 120 patients in the U.S., Hungary, and Australia (Shah et al., 2017a) **[NCT 02864381]**.

Finally, in Japan, the safety and efficacy of andecaliximab (800 mg, IV) as monotherapy will be compared with that of its combination with S1 (gimeracil/oteracil/tegafur) in 18 patients with advanced-stage GC^9^ **[NCT 02862535]**.

## Targeting gastric stem cell signaling pathways

### Characteristics of cancer stem cells (CSCS)

CSCs are typically not affected by conventional chemotherapy and radiation because of the presence of drug efflux pumps and efficient mechanisms for rapid DNA repair that allow these cells to escape apoptosis (Adams and Strasser, 2008; Bekaii-Saab and El-Rayes, 2017). Moreover, chemotherapy and radiotherapy exposure promote the accumulation of CSCs through several mechanisms, such as induction of the expression of *NOTCH1*, sonic hedgehog gene (*SHH*), and natural selection (Han et al., 2013). In addition, residual CSCs may account for tumor recurrence and metastases.

Gastric CSCs express certain distinct markers, such as CD44, CD44 variant (CD44v), EpCAM, CD24, CD133, and spheroid side population cells that facilitate identification and are useful for prognosis (Nguyen et al., 2016). Although, CD44 is present in up to 80% of resected specimens the expression of CD44v is more specific to patients with GC (Lau et al., 2014). The expression of CD44, CD44v, and CD133 has been shown to represent independent predictors for lower DFS and OS rates. In a meta-analysis, CD44 expression was associated with advanced-stage tumors (HR = 2.05; 1.12–3.75; *p* = 0.002) and lymph node metastases (HR = 1.5; 1.4–1.98; *p* = 0.004). The expression of CD44v was also associated with lymph node metastases (HR = 2.26; 1.4–3.64; *p* = 0.0008) and lymphovascular invasion (HR = 1.45; 1.05–2.01; *p* = 0.02), but not with progression to advanced stages (HR = 0.68; 0.36–1.28; *p* = 0.23; Wang et al., 2014).

It is likely that bone marrow-derived mesenchymal stem cells (BMDMSCs) that differentiate in the gastric mucosa by fusing with epithelial cells are a source of gastric CSCs. As a result of chronic inflammation caused by *Helicobacter pylori* infection (Brungs et al., 2016), these cells undergo epithelial-mesenchymal transformation (EMT). The down-regulation of E-cadherin (an epithelial marker) and up-regulation of N-cadherin, vimentin, and fibronectin (mesenchymal markers) enables epithelial cells to acquire mesenchymal traits, facilitating the invasion of local and distant sites (Dragu et al., 2015). EMT induces a stem cell-like state in differentiated cells (Dragu et al., 2015). Several other mechanisms have been proposed to explain the induction of stemness by *H. pylori*, including NANOG and octamer-binding transcription factor 4 (OCT4) up-regulation via Wnt/β-catenin signaling, cytotoxin-associated gene A (CagA)-mediated shatterproof 2 (SHP2) dysfunction, and inhibition of SHH signaling (Yong et al., 2016).

### Stemness inhibitors

**Figure 5:**
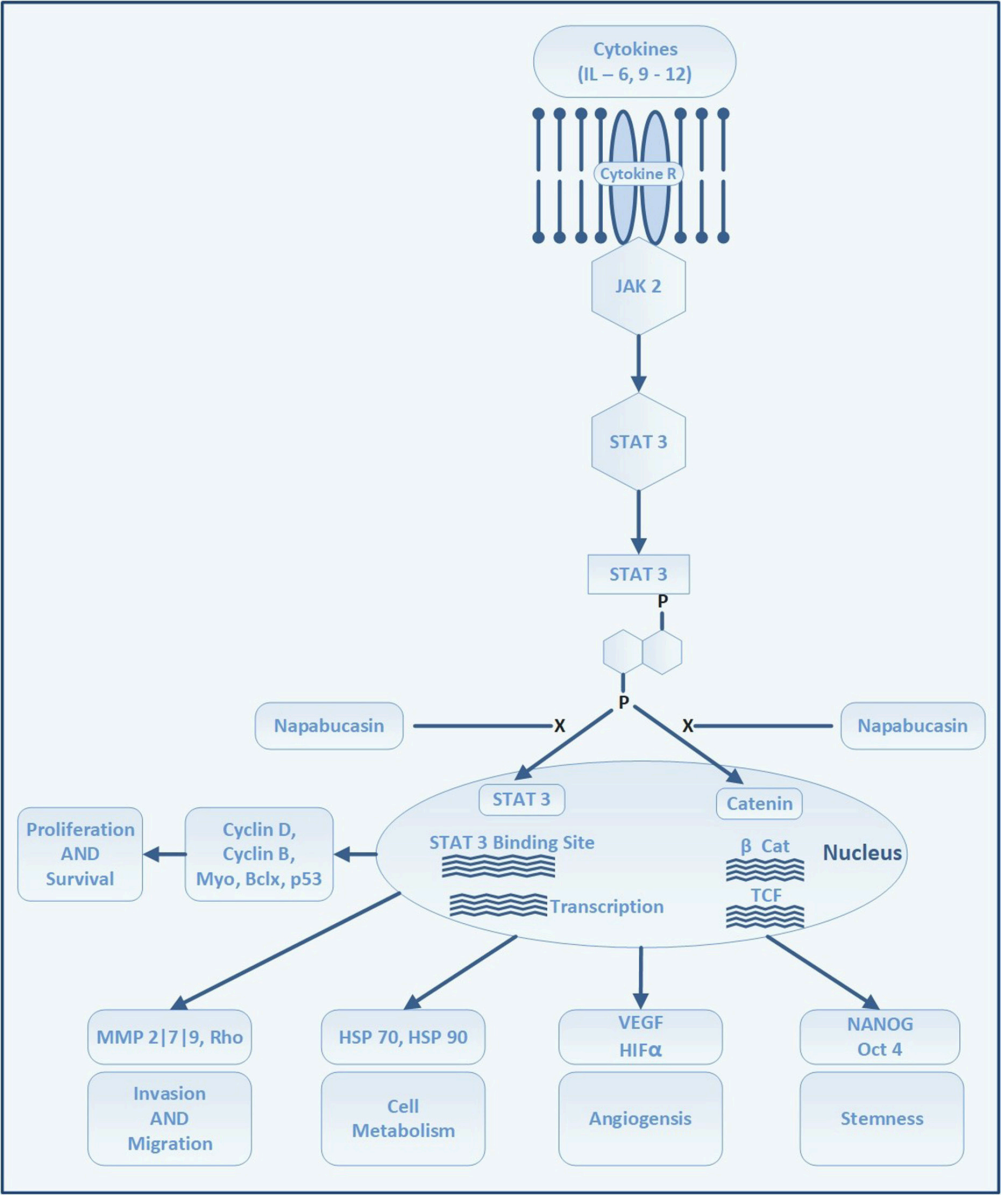
STAT 3 signaling pathway and site of action of napabucasin. Attachment of cytokines to its receptors activates Janus kinases which in turn phosphorylates STAT 3. The phosphorylation of STAT 3 leads to dimerization and activation of STAT 3. STAT 3 enters the nuclei to bind at STAT 3 binding sites on DNA which triggers transcription of several proteins which regulate cell proliferation, survival, angiogenesis, invasion and migration. Through Beta-catenin pathway STAT 3 induces genes responsible to trigger the formation and maintenance of stem cells.

Stemness is defined by the ability of cells to self-renew and differentiate into pluripotent cells. It is characterized by the expression of genes for stemness, such as *NANOG, OCT4*, and *SOX2* (Adams and Strasser, 2008). Among various pathways driving the stemness of gastric CSCs, a major therapeutic target is the signal transducer and activator of transcription 3 (STAT3) pathway (Figure 5). STAT3 is an important signaling pathway in the pathogenesis of GC and maintenance of the GC stem cell pool (Kamran et al., 2013). STAT3 was initially identified as a mediator of inflammation. In the physiological state, it maintains homeostasis and is only transiently active. Constitutive activation is noted in several cancer types, including breast, pancreatic, ovarian, prostate, and stomach cancers, as well as in leukemia (Aggarwal et al., 2009). Activation of STAT3 portends a poor prognosis because, together with *NANOG*, it preserves stemness characteristics, invasiveness, and tumorigenesis of cancer stem cells. Further, it regulates cell proliferation, invasion, migration, up-regulation of PD-L1, and angiogenesis in tumor microenvironments.

Although several approaches to targeting gastric CSC are being tested, the inhibition of STAT3 by napabucasin (formerly BBI608, developed by Boston Biomedical, acquired by Sumitomo Dainippon Pharma in 2012), which has shown promise in early phase trials of patients with advanced-stage GC, is currently being evaluated in a phase III trial. Napabucasin is an orally-administered first-in-class small molecule inhibitor of cancer stemness. It inhibits self-renewal of CSCs and induces CSC death by inhibiting the STAT3, β-catenin, and NANOG pathways (Li et al., 2015).

### Napabucasin (BBI608)

The safety of napabucasin in combination with paclitaxel was studied in a phase I study on Japanese patients (Shitara et al., 2015). Six patients with GC enrolled in the study received napabucasin (480 mg, twice daily) in combination with paclitaxel (80 mg/m^2^) weekly, for 3 weeks of each 28 days study cycle. The combination of napabucasin and paclitaxel was well tolerated by most patients in the study, and showed promising efficacy Table 1 [**JapicCTI 142420**].

The safety of escalating doses of napabucasin and weekly paclitaxel was also tested in a basket trial of 24 patients with a variety of solid tumors (Hitron et al., 2014); five of the patients had GC/GEC. The patients received napabucasin in escalating doses in combination with paclitaxel as described in Table 1. The combination of napabucasin and weekly paclitaxel was safe and elicited antitumor activity against several tumor types, including in patients with GC and GEC Table 1 [NCT 01325441].

In a phase Ib/II extension study, 46 patients with advanced GC/GEC received napabucasin combined with paclitaxel (Becerra et al., 2015; Table 1). Of the 46 patients, response to treatment was evaluated in 19 patients who had received taxane and 16 patients who had no prior taxane treatment (35 patients in total). The results further reinforced the notion that the combination was safe and well tolerated in patients with advanced GC/GEC, and therapeutic activity was maintained even in heavily pretreated patients **[NCT 01325441]**.

Following the encouraging results of the early phase trials, napabucasin is now undergoing a phase III trial (BRIGHTER), BBI608 plus weekly paclitaxel, to treat GC/GEC in patients who had received only one line of prior therapy (Shah et al., 2015). BRIGHTER is a randomized, double-blind, placebo-controlled trial that will enroll patients who have failed to respond to one previous line of therapy that involved fluoropyrimidine/platinum for advanced GC/GEC. Patients will be randomized to receive napabucasin, or placebo, twice daily in combination with weekly paclitaxel. The primary endpoint would be OS in all patients, while secondary endpoints will include safety, PFS, ORR, and DCR in all patients, and OS and PFS in a biomarker (β-catenin)-enriched subpopulation. In this trial, the survival benefit of adding napabucasin to paclitaxel compared with that for paclitaxel alone, and the efficacy of combination therapy in a biomarker-selected patient population will be determined **[NCT 02178956]**. At the time of writing the current review, an interim analysis conducted by an independent data and safety monitoring board (DSMB) suggested that the study is unlikely to meet its primary OS endpoint^10^. Following DSMB recommendations, sponsors unblinded this trial. Another stem cell inhibitor, vismodegib (which targets the Hedgehog pathway), has already failed to show any benefit in a phase II trial of patients with advanced GC (Cohen et al., 2013) **[NCT 00982592]**. However, patients who were CD44-positive showed a better response than those who were CD44-negative, suggesting the possible utility of vismodegib in patients with GC exhibiting high expression of stem cell biomarkers.

## Other therapeutic options

### Poly adenosine diphosphate ribose polymerase (PARP) inhibitors

Chromosomal instability is the hallmark of the chromosomal instability subtype (CIN) of GC according to TCGA classification (Network, 2014). A defect in DNA repair may lead to the loss of chromosomes associated with sensitivity to PARP inhibitors (Kubota et al., 2014). Previously, this had been demonstrated in ovarian cancer using rucaparib (Shapira-Frommer et al., 2015). In preclinical studies, the PARP inhibitor olaparib was active against GC cell lines with low levels of ataxia telangiectasia mutated (ATM) kinase, which is an activator of DNA damage response (Kubota et al., 2014). The benefit of combining olaparib with paclitaxel as a 2L treatment for recurrent or metastatic GC was shown in a phase II trial, but could not be confirmed in a subsequent phase III trial (Bang et al., 2015, 2017). However, these trials involved only few patients with low levels of ATM. A trial evaluating the safety and activity of olaparib in combination with durvalumab in patients with advanced-stage GC is ongoing **[NCT 02734004]**.

### Therapies targeting the her2-neu receptor

Trastuzumab in combination with chemotherapy is the 1L treatment for patients with HER2-positive GC/GEC (Ajani et al., 2016; Smyth et al., 2016; Association, 2017). Besides inhibiting HER2-neu, trastuzumab also leads to ADCC by interacting with a stimulatory receptor on NK cells and macrophages, CD16A FcγRIIIA. This stimulatory receptor is encoded by two alleles with different codons for amino acid 158: a V (valine) variant and an F (phenylalanine) variant, which are high- and low affinity variants, respectively (Nordstrom et al., 2011; Bang et al., 2017c). In breast carcinoma, the activity of trastuzumab is lower among patients who carry the low-affinity F allele than in patients who are homozygous for the V allele (Musolino et al., 2008). Moreover, loss of HER2-neu or ERBB2 amplification have been reported among patients with GC/GEC after treatment with trastuzumab (Pietrantonio et al., 2016). Margetuximab (MGAH22-10) is a novel HER2-neu–inhibiting monoclonal antibody that carries an Fc domain with enhanced affinity for the activating CD16A (FcγRIIIA) receptors on NK cells and monocytes, and reduced affinity for the inhibitory CD 32B (FcγRIIB) receptors, as compared with trastuzumab (Nordstrom et al., 2011). It was designed to be active irrespective of the CD 16A genotype. The combination of pembrolizumab and margetuximab was tested in a phase 1b/2 trial on patients with relapsed/refractory advanced HER2-neu^+^ PD-L1-unselected GC/GEC who failed trastuzumab therapy (Table 1; Catenacci et al., 2018). Equal number of Asian and non-Asians were enrolled in this trial. The preliminary results from this trial suggest that the combination of margetuximab and pembrolizumab exert antitumor activity against advanced GC/GEC as 2L treatment and is well tolerated. Better response was seen among patients with GC than ones with GEC, and in Asians than in non-Asian subjects. The rate of retention of ERBB2 amplification after margetuximab treatment was higher in GC than GEC. This could explain the observed higher response rate in patients with GC. Further, the GC proportion was higher in Asians than in non-Asians. These encouraging results were presented recently but complete data from this study are awaited **[NCT 02689284]**.

### Combined therapy with two her2-neu receptor antagonists

Although a significantly improved outcome was noted for the use of pertuzumab with trastuzumab and chemotherapy in HER2-positive breast cancer, this strategy is not successful in patients with metastatic GC/GEC. In a recently concluded phase III study, JACOB, 780 patients were randomized to receive pertuzumab, trastuzumab, and chemotherapy, or trastuzumab, chemotherapy, and placebo. Although the OS of patients in the pertuzumab arm was 3.3 months longer than that in the trastuzumab arm, it was not statistically different from that in the placebo arm (Tabernero et al., 2017; Table 1) **[NCT 01774786]**.

### Antibody-drug conjugate (ADC)

Another novel agent, DS-8201, is an anti-HER2 antibody-drug conjugate containing a linker and topoisomerase I inhibitor (with drug to antibody ratio of 7–8). It was tested in a phase I trial on heavily pretreated patients with HER2-positive GC (Iwasa et al., 2018). The study consisted of two parts, i.e., dose escalation (7 patients) and dose expansion (41 patients). The drug showed promising antitumor activity with ORR of 44% and DCR of 78% for patients in the second part of the study. Further, 83% patients experienced tumor shrinkage. The drug was also well tolerated, with the majority of patients developing only mild grade nausea. Nevertheless, 10% of patients discontinued the drug because of adverse effects. Although preliminary data appear to be promising, more data are needed before any conclusion is made [**NCT02564900**].

### Phosphatidylinositol 3-kinase (PI3K)/AkT/mTOR signaling pathway (everolimus, MK-2206, GSK2636771)

The PI3K/Akt/mTOR signaling pathway is vital for the regulation of the cell cycle. PI3K activation phosphorylates and activates AKT in the plasma membrane, which promotes cell proliferation and prevents apoptosis via several pathways, including mTOR. This pathway is antagonized by PTEN. Activation of the PI3/Akt/m-TOR pathway is associated with a relatively more invasive GC/GEC and nodal metastasis (Matsuoka and Yashiro, 2014). The role of drugs that target the PI3/Akt/m-TOR pathway is to restore the effectivity of chemotherapy against gastric cancer cells by inhibiting cell proliferation and supporting apoptosis (Matsuoka and Yashiro, 2014). However, significant efficacy of these agents was not demonstrated in clinical trials. In a phase III study (GRANITE-1), Everolimus (RAD001) failed to show survival benefits when compared with best supportive care (Ohtsu et al., 2013) **[NCT00879333]**. A phase II clinical trial of MK-2206 (AKT Inhibitor) was abandoned as a result of the failure of the drug to elicit survival advantage (Ramanathan et al., 2015) **[NCT01260701]**. Another agent, AZD5363, is a highly potent inhibitor of AKT, which competes with adenosine triphosphate (ATP) and inhibits all three isoforms of AKT with an inhibitory concentration of < 10 nmol/l. A phase II trial to evaluate AZD5363 in combination with paclitaxel as a 2L therapy for patients with advanced-stage GC/GEC harboring PI3KCA mutation or amplification is currently open **[NCT02451956]**. The safety and clinical activity of an oral PI3K-β inhibitor, GSK2636771, in combination with paclitaxel for patients with PTEN-deficient advanced-stage GC/GEC (as documented by alterations in the PI3K pathway genes, e.g., *PI3KCB, PI3KR1, PTEN*, etc., in fresh or archival tumor tissue) is being evaluated in a phase Ib/IIa dose-escalation trial. The study will be conducted in two phases. In the first phase (dose escalation, phase Ib), the maximum tolerated dose (MTD) and the recommended phase II dose (RP2D) of GSK2636771 in combination with paclitaxel will be determined. In the subsequent dose expansion phase (phase IIa), the safety and clinical activity of the RP2D (determined during the escalation phase) will be evaluated **[NCT02615730]**. GSK2636771 will be administered once daily (doses of 300 or 400 mg) with paclitaxel. In addition, two multi-arm phase II trials that also aim to study the safety and clinical activity of GSK2636771 in patients with PTEN-deficient advanced-stage GC/GEC (PTEN score less than 100) are currently recruiting patients **[NCT02951091 and NCT02465060**].

## Future directions

The genomic data from TCGA and ACRG classifications should be used to identify markers for responsive or resistant disease to enable future clinical trials to be organized to include biomarker-enriched patient populations. The use of biomarkers beyond PD-L1 expression, such as mononuclear density scores and INF-γ composite scores (previously utilized for melanoma), should be implemented routinely in ICI trials on patients with GC/GEC. Furthermore, the role of ICIs in the treatment of GC/GEC must be refined by the use of more extensive datasets and harmonization of PD-L1 assays. Data on the quality of life must be collected and reported on a regular basis, as most of the patients are heavily pretreated and their quality of life may be compromised by the accumulated toxicity. Long-term follow-up data from ICI trials should provide better insights into the safety profile of these agents in patients with GC/GEC.

## Conclusion

Ongoing GC/GEC studies have demonstrated a benefit from targeting tumor microenvironments with ICIs, claudiximab, and andecaliximab, as well as from targeting specific signaling pathways with napabucasin to modulate GC cell stemness. Most trials have been directed toward immunotherapy, especially ICIs as a single-agent therapy or in combination with chemotherapy. The IHC data that are collected as part of ongoing trials should further our understanding of the impact of PD-1/PD-L1 expression on the outcome of advanced GC. Despite these advances, the absolute gains have been modest, with a longevity increase of few months achieved with most of these agents. Therefore, the identification of more actionable novel targets is warranted to increase the treatment spectrum for patients with advanced-stage GC/GEC. Additionally, further studies aimed at the identification and validation of biomarkers to accurately identify patients who may derive benefit from anticancer agents should be prioritized.

## Author contributions

VK: original idea, literature search, manuscript writing editing, proofreading; MG, PS: literature search, manuscript writing editing, proofreading; SK, AC: editing, proofreading.

### Conflict of interest statement

The authors declare that the research was conducted in the absence of any commercial or financial relationships that could be construed as a potential conflict of interest.

## Footnotes

^1^2017f. GLOBOCAN Cancer Fact Sheets: stomach Cancers.

^2^2018. *Stomach Cancer - Cancer Stat Facts* [Online]. Available: https://seer.cancer.gov/statfacts/html/stomach.html [Accessed].

^3^KEYNOTE-061 2017. *ClinicalTrial; A Study of Pembrolizumab (MK-3475) Versus Paclitaxel for Participants With Advanced Gastric/Gastroesophageal Junction Adenocarcinoma That Progressed After Therapy With Platinum and Fluoropyrimidine (MK-3475-061/KEYNOTE-061) (2017)- Full Text View - ClinicalTrials.gov* [Online]. Available: https://clinicaltrials.gov/ct2/show/NCT02370498 [Accessed].

^4^Press Release 2018. Pembrolizumab Misses Endpoints in Phase III Gastric Cancer Trial.

^5^KEYNOTE-062 2017. *KEYNOTE-062. Study of Pembrolizumab (MK-3475) as First-Line Monotherapy and Combination Therapy for Treatment of Advanced Gastric or Gastroesophageal Junction Adenocarcinoma (MK-3475-062/KEYNOTE-062) - Full Text View - ClinicalTrials.gov* [Online]. Available: https://clinicaltrials.gov/ct2/show/NCT02494583 [Accessed].

^6^ClinicalTrials.gov, 2018. *A Study of IMU-131 Plus Standard of Care Chemotherapy in Patients With HER2/Neu Overexpressing Advanced Cancer of the Stomach - Full Text View - ClinicalTrials.gov* [Online]. Available: https://clinicaltrials.gov/ct2/show/NCT02795988 [Accessed].

^7^2017e. *Claudiximab - AdisInsight* [Online]. Available: http://adisinsight.springer.com/drugs/800030310 [Accessed].

^8^2017a. *Andecaliximab - AdisInsight* [Online]. Available: http://adisinsight.springer.com/drugs/800038052 [Accessed].

^9^2017b. *Andecaliximab as Monotherapy and in Combination With Anti-Cancer Agents in Japanese Participants With Gastric or Gastroesophageal Junction Adenocarcinoma - Full Text View - ClinicalTrials.gov* [Online]. Available: https://clinicaltrials.gov/ct2/show/NCT02862535 [Accessed].

^10^BOSTON BIOMEDICAL, I. 2018. *Boston Biomedical Announces Unblinding of the Phase 3 Trial in Patients with Advanced Gastric/GEJ Cancer* [Online]. @PRNewswire. Available: https://www.prnewswire.com/news-releases/boston-biomedical-announces-unblinding-of-the-phase-3-trial-in-patients-with-advanced-gastricgej-cancer-300479437.html [Accessed].
